# Peripheral Immune Cell Gene Expression Changes in Advanced Non-Small Cell Lung Cancer Patients Treated with First Line Combination Chemotherapy

**DOI:** 10.1371/journal.pone.0057053

**Published:** 2013-02-25

**Authors:** Yung-Che Chen, Chang-Chun Hsiao, Kuang-Den Chen, Yu-Chiang Hung, Ching-Yuan Wu, Chien-Hao Lie, Shih-Feng Liu, Ming-Tse Sung, Chung-Jen Chen, Ting-Ya Wang, Jen-Chieh Chang, Petrus Tang, Wen-Feng Fang, Yi-Hsi Wang, Yu-Hsiu Chung, Tung-Ying Chao, Sum-Yee Leung, Mao-Chang Su, Chin-Chou Wang, Meng-Chih Lin

**Affiliations:** 1 Division of Pulmonary and Critical Care Medicine, Kaohsiung Chang Gung Memorial Hospital and Chang Gung University College of Medicine, Kaohsiung, Taiwan; 2 Center of Translational Research in Biomedical Sciences, Kaohsiung Chang Gung Memorial Hospital and Chang Gung University College of Medicine, Kaohsiung, Taiwan; 3 Department of Chinese Medicine, Kaohsiung Chang Gung Memorial Hospital and Chang Gung University College of Medicine, Kaohsiung, Taiwan; 4 Department of Respiratory Therapy, Kaohsiung Chang Gung Memorial Hospital and Chang Gung University College of Medicine, Kaohsiung, Taiwan; 5 Department of Pathology, Kaohsiung Chang Gung Memorial Hospital and Chang Gung University College of Medicine, Kaohsiung, Taiwan; 6 Division of Rheumatology, Kaohsiung Chang Gung Memorial Hospital and Chang Gung University College of Medicine, Kaohsiung, Taiwan; 7 Department of Medical Research, Kaohsiung Chang Gung Memorial Hospital and Chang Gung University College of Medicine, Kaohsiung, Taiwan; 8 Graduate Institute of Clinical Medical Sciences, Chang Gung University College of Medicine, Kaohsiung, Taiwan; Roswell Park Cancer Institute, United States of America

## Abstract

**Introduction:**

Increasing evidence has shown that immune surveillance is compromised in a tumor-promoting microenvironment for patients with non-small cell lung cancer (NSCLC), and can be restored by appropriate chemotherapy.

**Methods:**

To test this hypothesis, we analyzed microarray gene expression profiles of peripheral blood mononuclear cells from 30 patients with newly-diagnosed advanced stage NSCLC, and 20 age-, sex-, and co-morbidity-matched healthy controls. All the patients received a median of four courses of chemotherapy with cisplatin and gemcitabine for a 28-day cycle as first line treatment.

**Results:**

Sixty-nine differentially expressed genes between the patients and controls, and 59 differentially expressed genes before and after chemotherapy were identified. The *IL4* pathway was significantly enriched in both tumor progression and chemotherapy signatures. *CXCR4* and *IL2RG* were down-regulated, while *DOK2* and *S100A15* were up-regulated in the patients, and expressions of all four genes were partially or totally reversed after chemotherapy. Real-time quantitative RT-PCR for the four up-regulated (*S100A15, DOK2*) and down-regulated (*TLR7, TOP1MT*) genes in the patients, and the six up-regulated (*TLR7, CRISP3, TOP1MT*) and down-regulated (*S100A15, DOK2, IL2RG*) genes after chemotherapy confirmed the validity of the microarray results. Further immunohistochemical analysis of the paraffin-embedded lung cancer tissues identified strong S100A15 nuclear staining not only in stage IV NSCLC as compared to stage IIIB NSCLC (p = 0.005), but also in patients with stable or progressive disease as compared to those with a partial response (p = 0.032). A high percentage of S100A15 nuclear stained cells (HR 1.028, p = 0.01) was the only independent factor associated with three-year overall mortality.

**Conclusions:**

Our results suggest a potential role of the *IL4* pathway in immune surveillance of advanced stage NSCLC, and immune potentiation of combination chemotherapy. S100A15 may serve as a potential biomarker for tumor staging, and a predictor of poor prognosis in NSCLC.

## Introduction

Non-small cell lung cancer (NSCLC) is the most common cause of cancer-related deaths worldwide. The average 5-year survival rate is less than 15%, which has remained largely unchanged for the last three decades. The majority of NSCLC patients present with advanced disease at diagnosis, and those diagnosed with early stage disease often experience recurrence and metastatic disease [1], [2], [3].

Host immune cells mediate immune surveillance by eradicating aberrant cells, and this is compromised in a tumor-promoting microenvironment for many patients with lung cancer. Several immune defects, including a shift toward the type 2 helper T cell (Th2) phenotype, are evident in lung cancer patients [4], [5]. However, the same immune cells may promote tumor growth and metastasis through angiogenesis and invasion of the extracellular matrix [6], [7]. Understanding the fundamental molecular processes that cause these defects would provide an opportunity to develop innovative therapies. In addition, immune cell responses mounted by various histopathological types and tumor stages of lung cancer may be different; however, studies on this issue are lacking.

Several immunosuppressive molecules are produced by tumors, such as interleukin-10 (IL-10), transforming growth factor-beta (TGF-β), or cyclooxygenase-2 (COX-2) metabolites; however, specific therapies such as chemotherapy and radiotherapy may contribute to the alteration of immune system function [4], [6], [8]. Increasing evidence suggests that a facet of immune surveillance can be restored by appropriate chemotherapy agents. For example, the nucleoside analogue gemcitabine (GEM) has been shown to selectively enhance the adaptive immune response and promote the cell-mediated immune response over the humoral immune response in addition to conventional apoptotic effects [9]–[12]. In addition, the platinum-based agent, cisplatin (CDDP), has been shown to augment the anti-tumor effects of cytotoxic T-lymphocyte-mediated immunotherapy [13]. It has also been demonstrated that using platinum-based double chemotherapy yields a significant benefit in terms of tumor response and survival compared with a single-agent regimen [14]. The underlying mechanism of immune potentiation for combination chemotherapy is largely unknown.

The aim of this study was to improve the understanding of the molecular mechanisms that regulate immunosurveillance or tumor progression in the immune cells of patients with advanced stage NSCLC by investigating the expressions of genes in peripheral blood mononuclear cells (PBMC) that may be involved in these effects. We hypothesized that the gene expressions of PBMC involved in the immune response to advanced stage NSCLC would be markedly different from those in healthy subjects, and that additional differences would be seen between cancer patients with adenocarcinoma (AC) and squamous cell carcinoma (SCC) or between stage IIIB and IV. Furthermore, we aimed to improve the understanding of the molecular mechanisms that regulate immunopotentiation induced by combination chemotherapy with CDDP and GEM, with the hope that novel genes may be found to be over- or under-expressed after treatment, thus offering new insights into improving the efficacy of chemotherapy.

A number of studies have applied DNA microarray technology to investigate gene expressions in patients with NSCLC [15]–[24]. In one of these studies, which focused on gene expressions in the blood leukocytes rather than tumor tissues, 29 genes were found to be altered in patients with early-stage NSCLC compared to those with non-malignant lung conditions. The extent to which the leukocyte genes play a role in advanced NSCLC, and the effects of histopathology and tumor stage on gene signatures are unclear [23]. Therefore, we extended our investigation into advanced-stage NSCLC by analyzing whole-genome gene expression profiles in PBMC from patients with newly-diagnosed advanced stage NSCLC and histopathology of either AC or SCC. Furthermore, to establish a direct link between gene expression and chemotherapy, post-treatment PBMC from 17 patients who received at least four courses of combination chemotherapy with CDDP and GEM were obtained, and the effects of chemotherapy on global gene expression profiles were evaluated using microarray analysis.

## Materials and Methods

The study was approved by the Institutional Review Board of Chung Gung Memorial Hospital, Taiwan. The study participants were recruited from the pulmonary clinics and health examination center of Kaohsiung Chung Gung Memorial Hospital during the period August 2007 through January 2009. All of the enrolled patients were aged 18 years or older with histologically confirmed, newly diagnosed, untreated, stage IIIB or IV NSCLC, with one or more measurable (unidimensional) lesion with a diameter of 10 mm or larger measured using computerized tomography, histopathologic subtype of AC or SCC, and an Eastern Cooperative Oncology Group performance status of 0, 1, or 2. Each tumor was diagnosed according to the histopathologic subtype and grade by a pathologist. Clinical stage was judged according to the International Union Against Cancer (UICC) Tumor Node Metastasis classification (6^th^ edition, 2002). A total of 42 patients with lung cancer were screened for eligibility of enrollment. Patients with first line treatment regimens other than CDDP and GEM combination chemotherapy (4 patients), those receiving concurrent radiotherapy (2 patients), serious uncontrolled medical or psychiatric illness (1 patient), history of other malignancy within the last 5 years (1 patient), and refusing to provide a blood sample for the study (4 patients) were excluded. Thirty patients completed follow-up and were included for final analysis. All 30 patients received first line chemotherapy with CDDP (mean dose 70 mg/m2) on day 1 plus GEM (GEMZAR®; Eli Lilly and Company, Indianapolis, IN; mean dose 1000 mg/m2) on day 1, 8, and 15 of a 28-day cycle. Treatment was planned for a maximum of six cycles (median 4, range 1–6) unless it was discontinued because of progressive disease, stable disease, unacceptable toxicity, or death. After baseline evaluation, tumor response was assessed one week after the last dose according to the Response Evaluation Criteria in Solid Tumors. A complete response (CR) was defined as the complete disappearance of all evidence of disease. A partial response (PR) was defined as a reduction of at least 30% of the largest diameter of each target lesion, without the appearance of new lesions. Progression was defined as an increase of at least 20% of the largest diameter of each target lesion, or the appearance of one or more new lesions. Overall survival was defined as time from diagnosis until death from any cause in the 3-year follow-up period. Twenty healthy subjects without a history of malignancy within the past 5 years or recent infectious disease were enrolled as the control group.

### Processes of RNA Isolation and cRNA Synthesis

Peripheral whole blood (15 ml) was collected after informed consent had been obtained from the 30 patients with newly-diagnosed histopathologically confirmed NSCLC, and the 20 age-, sex-, and co-morbidity-matched healthy controls (HC). Another 15 ml of blood was collected again one week after the last dose of CDDP and GEM from the 17 patients who completed at least four courses of combination chemotherapy. The PBMC were isolated by Ficoll-Hypaque gradient centrifugation (HISTOPAQUE®-119, Sigma-Aldrich, Inc., St. Louis, MO USA) within 90 min of drawing blood, washed in PBS, and then stored in RNAlater (Ambion Inc., Austin, TX, USA) at −80°C until RNA isolation. An RNeasy® Plus Mini Kit (Qiagen, Hilden, Germany) was used for isolation of high quality total RNA, and treated with DNase according to the manufacture protocol. RNA was then eluted in RNase-free water. Samples were run on a RNA 6000 Nano Gel System (Agilent Technologies Inc., Palo Alto, CA, USA) using an Agilent 2100 Bioanalyzer (Agilent) to determine the quality of RNA, and 2 ul RNA was used to determine the concentration of RNA using a NanoDrap spectrophotometer (Thermo Science, Wilmington, DE, USA). Only samples with A260/A280 ratios of 1.9 to 2.1 were used for further analysis. A total of 300 ng RNA was used for synthesis of first strand cDNA and in vitro transcription of cRNA using an Illumina Totalprep RNA Amplication kit (Ambion, Inc.).

### Gene Expression Profiling and Microarray Data Analysis

Illumina (San Diego, CA) HumanRef-8v2 bead microarrays were used with 750 ng labeled cRNA for each sample according to the manufacturer’s protocol. Human Ref-8 version 2 arrays consist of 22,184 probes representing 18,189 unique human genes on an eight-strip format array. All expression dataset has been deposited in the NCBI Gene Expression Omnibus (GEO) with accession number GSE39345. Statistical analysis of the microarray data was performed, using GeneSpring software version 11 (Agilent Technologies Inc., Santa Clara, CA, USA). To make the expression values independent of spot intensity and location, a further per gene normalization was applied to the pre-normalized data within each array by dividing the raw data by the median of the expression level for the gene in samples from healthy subjects. Raw data generated after the scanning of the microarrays with background corrected were loaded from Illumina’s Beadstudio Expression module to Genespring GX. This step was followed by quantile normalization and log2 transformation. A probe set filter was applied to remove genes that were not reliably detected. Overall, 22150 probes of the total 22184 probes (99.8%) showed where at least 1 out of 67 samples had been flagged in P (present) or M (marginal) flags compared to background levels. A non-parametric U test for unpaired comparisons of the two independent groups or the Wilcoxon signed rank test to compare gene expression levels before and after chemotherapy was applied. We used Benjamini-Hochberg false-discovery rate (FDR) correction method controlling for false positives and a corrected p-value cutoff of 0.05 was used to select the sets of significantly up- and down-regulated genes with the lowest FDR. The gene sets were then grouped into functional categories according to the Gene Ontology Biological Processes Classification. The pathway enrichment process was used to identify the features from significantly altered subsets of genes among phenotypic groups and find direct relationships between genes of interest. This was performed in GeneSpring software with the “simple and direct interaction” algorithm. The information used by the algorithm was obtained from the PubMed literature using Natural Language Processing algorithms to search keywords and relationships in abstracts. Hierarchical clustering analysis was performed by clustering and tree-building programs, based on the filtered genes that met the statistical criteria, using the hexagonal bubble method.

### Verification of Gene Expressions using TaqMan Quantification Real-time Reverse Transcriptase-polymerase Chain Reaction (RT-PCR)

To confirm the expression patterns of certain candidate genes, the expressions were analyzed using RT-PCR in a 32-well format. For each gene of interest, a single RT-PCR reaction was performed on the same RNA sample of the PBMC from the cancer patients (N = 30) and HC (N = 20). The house keeping gene Human acidic ribosomal protein (HuPO) was chosen as an endogenous control to normalize the expression data for each gene. All PCR primers (random hexamers) and TaqMan probes were designed and purchased from Roche according to the company’s protocols (www.roche-applied-science.com), and their sequences are given in Table S1, which is published as supporting information at the PLoS One web site. Briefly, two-step quantitative RT-PCR was performed. First strand cDNA was synthesized from 5 µg of RNA using a LightCyclerTaqManMaster kit (Roche, Germany). The PCR reactions were run in a Roche Light Cycle 2.0 machine. Single real time PCR experiment was carried out on each sample for each target gene, because the Roch Light Cycler system has shown high reproducibility using excellent thermal control and probe detection format (https://www.roche-applied-science.com/sis/rtpcr/LCNano/index.jsp?id=LCNano_100000). Relative expression levels were calculated using the ΔΔCq method with the median value for the HC group as the calibrator, as was done with microarray data [25].

### Immunohistochemistry (IHC) Staining of Lung Cancer Tissues for S100A15

Tissue paraffin sections were deparaffinized in xylene, rehydrated through graded ethanol solutions to distilled water, and then washed in Tris-buffered saline. Antigen retrieval was carried out by heating sections in 10 mM sodium citrate for 10 min in a microwave oven. Endogenous peroxidase activity was quenched by incubation in 3% H_2_O_2_ in methanol for 5 min. Non-specific binding sites were blocked using Protein Block (Dako, Carpinteria, California, USA) for 20 min. The tissue sections were then incubated for 1 h at room temperature with anti-human S100 calcium binding protein A15 (anti-hS100A15;1∶5000; a gift from Stuart H. Yuspa, Laboratory of Cancer Biology and Genetics Center for Cancer Research, National Cancer Institute, USA) overnight, followed by 30 min-incubation with goat anti-mouse IgG conjugated to a horseradish peroxidase-labeled polymer (Envision+ System; DakoCytomation, Carpinteria, California, USA). Slides were developed for 5 min with 3,3′-diaminobenzidine chromogen and counterstained with haematoxylin. In the negative control tissue sections, the primary antibody was replaced by isotype specific non-immune mouse IgG.

### Evaluation of Immunohistochemical Staining for S100A15

Each slide was evaluated for S100A15 immunostaining using a semi-quantitative scoring system for both staining intensity and the percentage of positive tumor/pulmonary epithelial cells. IHC staining overview was performed by an independent trained reader (TYW), who was blinded to the clinical outcome, tumor stage, and histopathology. The tissue sections were scored by manually counting ≥1000 cells based on the percentage of immunostained cells as: 0–10% = 0; 10–30% = 1; 30–50% = 2; 50–70% = 3 and 70–100% = 4. Sections were also scored semi-quantitatively on the basis of staining intensity as negative = 0; mild = 1; moderate = 2; and intense = 3. A total score was obtained by adding the scores of percentage positivity and staining intensity, with nuclear and cytoplasmic staining scored independently [26].

### Statistical Analysis

Continuous values were presented as the mean ± standard deviation (SD). Mann-Whitney, Wilcoxon ranked sum, Kruskal-Wallis H, and chi-square tests were used to assess the differences between different groups where appropriate. Pearson correlation test was used to assess the correlation between two continuous variables. The Kaplan-Meier method was used to estimate overall survival, and Cox regression analysis with stepwise forward selection was used to evaluate independent prognostic factors associated with survival. All tests were two tailed and the null hypothesis was rejected at p<0.05. A statistical software package (SPSS, version 15.0, SPSS Inc., Chicago, IL) was used for all analyses.

## Results

Microarrays from the 50 subjects that passed the quality-control filters were included in this study. The demographic and clinical data of these subjects (30 patients with advanced stage NSCLC and 20 HC), are presented in Table 1. No significant differences were observed between the patients and HC with respect to age, sex, co-morbidity, and smoking history. The cancer patients included 9 stage IIIB AC, 12 stage IV AC, 5 stage IIIB SCC, and 4 stage IV SCC.

**Table 1:** Baseline and clinical characteristics of the 30 advanced stage non-small cell lung cancer patients and 20 matched healthy controls.

	Healthy subjectsN = 20	Cancer patientsN = 30	P value
Age, years	61.4±12.6	62±11.9	0.855
Male, n (%)	12 (60)	21 (70)	0.465
Co-morbidity, n (%)			
Hypertension	9 (45)	11 (36.7)	0.556
Diabetes mellitus	4 (20)	3 (10)	0.318
Chronic obstructive pulmonary disease	5 (25)	10 (33.3)	0.529
Heart failure	2 (10)	1 (3.3)	0.331
Chronic hepatitis	1 (5)	3 (10)	0.523
Chronic renal failure	3 (15)	2 (6.7)	0.336
Smoking history, n (%)			0.447
Never	8 (66.7)	15 (50)	
Remote	2 (16.7)	11 (36.7)	
Current	2 (16.7)	4 (13.3)	
Tumor Stage, n (%)			
IIIB		14 (46.67)	
IV		16 (53.33)	
Histopathology, n (%)			
Squamous cell carcinoma		9 (30)	
Adenocarcinoma		21 (70)	
ECOG performance status, n (%)			
0		3 (10)	
1		22 (73.3)	
2		5 (16.7)	
Response to first line chemotherapy, n (%)			
Partial response		9 (30)	
Stable disease		13 (43.3)	
Progressive disease		8 (26.7)	

ECOG = Eastern Cooperative Oncology Group.

### Differentially Expressed Genes in PBMC Associated with Tumor Progression

From the 22,150 probes, 4463 (20.16%) transcripts were differentially expressed between patient group and HC group using a Mann-Whitney unpaired test with a Benjamini-Hochberg FDR of <0.05. For the stage-dependent change in mRNA expression levels from patients of stage IIIB and IV comparing to HC group, a Kruskal-Wallis test with Benjamini-Hochberg FDR of <0.05 by a concomitant 1.5-fold increase was applied and 3989 differentially expressed transcripts were identified. Furthermore, 3858 of these transcripts intersected with those found in the Mann-Whitney test. Using the functional pathway categories of the GeneSpring pathway analysis tool as the selection criteria for further validation, the 3858 gene sets were then analyzed for functional annotation at the pathway level. Eleven statistically different functional categories were mapped, and 70 gene transcripts (69 unique genes) involved in the secretion, DNA topoisomerase activity, methyltransferase activity, histone acetyltransferase activity, growth factor activity, protein tyrosine phosphatase activity, membrane lipid metabolic process, innate immune response, cell adhesion molecule binding, metalloendopeptidase inhibitor activity, and cytokine/chemokine mediated signaling pathways were identified as putative signatures of these functions (Table S2).

### Differentially Expressed Genes in PBMC after Combination Chemotherapy with CDDP and GEM in Cancer Patients

To assess the effects of chemotherapy on peripheral immune cells, gene expression profiles in the seventeen patients who completed at least four courses of CDDP and GEM treatment were compared before and after chemotherapy using Mann-Whitney U test for paired observations (p<0.05), and differential expression of 3576 transcripts was identified. Additionally, a total of 669 transcripts were intersected between differentially expressed transcripts in paired cases upon chemotherapy and the 3858 transcripts identified with cancer/staging specificity. Using the pathway analysis tool in GeneSpring GX, nine functional categories were mapped, and 62 gene transcripts (59 unique genes) involving innate immune response, cytokine production, microtubule-based process, organic cation transmembrane transporter activity, secretory pathway, DNA topoisomerase (ATP-hydrolyzing) activity, histone methyltransferase activity, histone acetyltransferase activity, and protein tyrosine phosphatase activity were identified from this set of 669 gene transcripts (Table S3). Six genes with higher expressions with tumor progression showed even higher expressions after chemotherapy: *Cysteine-rich secretory protein 3* (*CRISP3*; innate immune response protein), *signal transducer and activator of transcription 1* (*STAT1*; cytokine and growth factor receptor signaling mediator), *Solute carrier family 22 member 4* (*SLC22A4*; ergothioneine transporter), *Synapsin II, transcript variant IIa* (*SYN2*; synaptic transmission), *Brain-derived neurotrophic factor* (*BDNF*; growth factor for survival and differentiation of neurons and synaptic transmission), *Potassium large conductance calcium-activated channel, subfamily M, alpha member 1* (*KCNMA1*; synaptic transmission, cell cycle, and cell migration). Notably, *S100A15* (also called *S100A7A;* epidermal maturation and antimicrobial defense) was up-regulated with tumor progression and reversed to normal after chemotherapy.

### Interleukin 4 (IL4) Pathway

**Figure 1:**
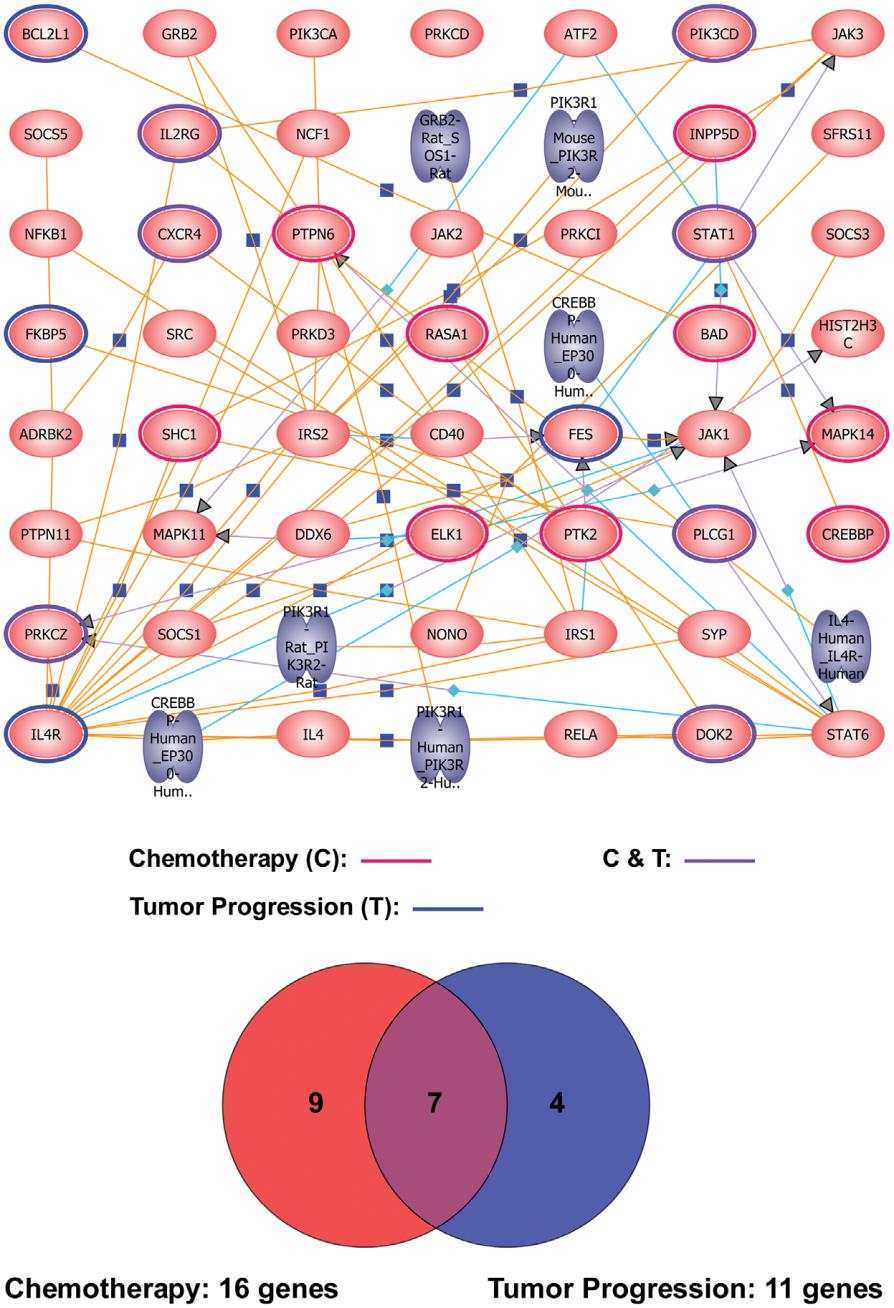
The *IL4* pathway was enriched in both tumor progression and chemotherapy signatures. Eleven out of the 49 *IL4* pathway-involved genes were mapped in the pathway enrichment analysis for the 3,858 differentially expressed transcripts in PBMC among patients with advanced non-small ell lung cancer (NSCLC) and normal controls, whereas 16 unique genes were mapped for the 669 differentially expressed transcripts in PBMC of patients with advanced-stage NSCLC before and after chemotherapy. Seven intersected genes between the two gene-sets were identified and presented in a Venn diagram. The blue circle indicates differentially expressed genes associated with tumor progression solely, the red circle with chemotherapy solely, and the purple circle with both effects.

We found 69 genes to be significantly differentially expressed between the patients and healthy controls, and 59 genes differentially expressed after chemotherapy. To investigate whether the genes were differentially expressed in advanced stage NSCLC and with chemotherapy in common pathways, we studied their connectivity using the GeneSpring pathway analysis tool. The *IL4* pathway was significantly enriched in both signatures of tumor progression and chemotherapy. Among the 49 genes involved in the IL4 pathway, 11 unique genes (12 gene transcripts) were differentially expressed between the patients and normal controls, and 16 unique genes (19 gene transcripts) were differentially expressed before and after chemotherapy. The intersection of both comparisons resulted in 7 unique genes (Figure 1 and Table 2). Notably, *chemokine C-X-C motif receptor 4* (*CXCR4*; 7-transmembrane G-protein chemokine receptor for *CXCL12*) was up-regulated, and *docking protein 2* (*DOK2*; the substrate of chmeric p210bcr/abl oncoprotein) and *interleukin 2 receptor, gamma* (*IL2RG*; interleukin receptor common gamma chain for *IL2, IL4, IL7, IL9, IL15*, and *IL21*) were down-regulated with tumor progression, while expressions of these three genes changed in the opposite direction after chemotherapy. On the other hand, *Phospholipase C, gamma 1* (*PLCG1*; guanine nucleotide exchange factor for nuclear GTPase), *Phosphoinositide-3-kinase, catalytic, delta polypeptide* (*PIK3CD;* autophosphorylation activity), and *Protein kinase C, zeta* (*PRKCZ*; atypical isozyme of the serine-threonine protein kinase C) which showed decreased expressions with tumor progression were further down-regulated with chemotherapy, while *STAT1* which showed an increased expression with tumor progression was further up-regulated with chemotherapy.

**Table 2:** IL4 pathway-associated genes that were differentially expressed with tumor progression and/or chemotherapy.

Gene Name	Fold Change			Gene Bank ID	Description
	Stage* IIIB	Stage* IV	C/T#		
IL2RG	2.631	1.950	0.437	NM_000206.1	Interleukin 2 receptor, gamma
STAT1	1.156	1.800	1.602	NM_007315.2	Signal transducer and activator of transcription 1, 91 kDa, transcript variant alpha
CXCR4	0.165	0.186	1.452	NM_003467.2	Chemokine (C-X-C motif) receptor 4, transcript variant 2
DOK2	1.585	1.500	0.719	NM_003974.2	Docking protein 2, 56 kDa
PLCG1	0.748	0.492	0.5	NM_182811.1	Phospholipase C, gamma 1, transcript variant 2
PIK3CD	0.764	0.690	0.626	NM_005026.2	Phosphoinositide-3-kinase, catalytic, delta polypeptide
PRKCZ	0.664	0.307	0.421	NM_002744.4	Protein kinase C, zeta, transcript variant 1
IL4R	0.426	0.241		NM_001008699.1	Interleukin 4 receptor, transcript variant 2
IL4R	0.748	0.587		NM_000418.2	Interleukin 4 receptor, transcript variant 1
FKBP5	1.475	1.407		NM_004117.2	FK506 binding protein 5
BCL2L1	2.207	1.938		NM_138578.1	BCL2-like 1, nuclear gene encoding mitochondrial protein, transcript variant 1
FES	2.081	2.402		NM_002005.2	Feline sarcoma oncogene
PRKCZ			2.468	NM_001033581.1	protein kinase C, zeta, transcript variant 2.
MAPK14			2.456	NM_139012.1	Mitogen-activated protein kinase 14, transcript variant 2.
PTK2			1.433	NM_005607.3	PTK2 protein tyrosine kinase 2, transcript variant 2.
BAD			2.964	NM_032989.1	BCL2-antagonist of cell death, transcript variant 2.
RASA1			2.348	NM_022650.1	RAS p21 protein activator (GTPase activating protein) 1, transcript variant 2.
CREBBP			0.756	NM_001079846.1	CREB binding protein (Rubinstein-Taybi syndrome), transcript variant 2.
PTPN6			0.657	NM_080548.3	Protein tyrosine phosphatase, non-receptor type 6 (PTPN6), transcript variant 2.
PTPN6			0.336	NM_080549.2	Protein tyrosine phosphatase, non-receptor type 6 (PTPN6), transcript variant 3.
SHC1			0.389	NM_183001.3	SHC (Src homology 2 domain containing) transforming protein 1 (SHC1), transcript variant 1.
SHC1			0.727	NM_003029.3	SHC (Src homology 2 domain containing) transforming protein 1 (SHC1), transcript variant 2.
INPP5D			0.794	NM_005541.3	Inositol polyphosphate-5-phosphatase, 145 kDa (INPP5D), transcript variant 2.
ELK1			0.40967	NM_005229.2	ELK1, member of ETS oncogene family.

* Gene expression levels of PBMC samples from stage IIIB or IV NSCLC patients compared to those from healthy controls.

# Gene expression levels of PBMC samples from NSCLC patients after chemotherapy (C/T) compared to those before C/T.

### Distinct Expression Patterns Across Tumor Stages and Histopathological Subtypes

**Figure 2:**
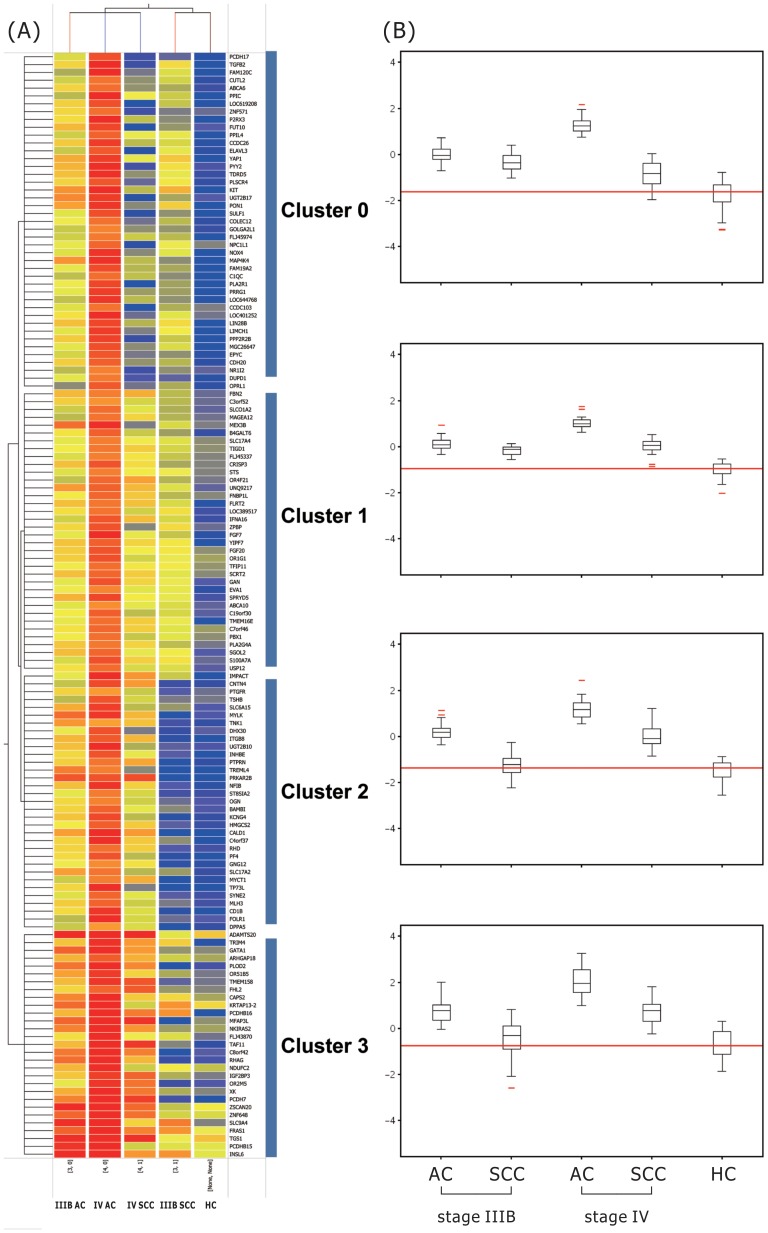
Hierarchical clustering of PBMC samples from patients with advanced stage lung cancer and healthy controls. (A) Hierarchical clustering of patients with newly-diagnosed advanced stage non-small cell lung cancer (n = 30) and healthy controls (n = 20) according to the expression of the 69 genes differentially expressed among stage IIIB adenocarcinoma (AC), stage IV AC, stage IIIB squamous cell carcinoma (SCC), stage IV SCC, and healthy controls (HC). The data are presented in a matrix format: each row represents a gene on the microarray and each column a subgroup of mRNA samples. The ratios of hybridization of fluorescent cDNA probes were a measure of relative gene expression in each experimental sample to a reference mRNA sample, and are depicted according to the following color scale: red indicates a high level of expression; blue, low level of expression; and yellow, mean level of expression. (B) Normalized median gene expression values for cluster 0–3 genes in five subject categories (stage IIIB AC, stage IIIB SCC, stage IV AC, stage IV SCC, HC). Note that cluster 1 genes showed higher expressions in all lung cancer patients compared to the HC. Cluster 0 genes showed similar expressions in stage IV SCC and HC. Both cluster 2 and 3 genes showed similar expressions in stage IIIB SCC and HC. The box plots show the 25^th^, 50^th^, and 75^th^ percentiles, maximum, and minimum.

To further clarify the effects of both tumor stage and histopathological subtypes on the gene signatures of PBMC, differentially expressed genes were clustered by using a hierarchical clustering algorithm with an average-linkage method and euclidean distance metric in Genespring GX. Figure 2 shows two dimensional hierarchical clustering of all the cancer patients and HC based on the 73 genes that were differentially expressed among stage IIIB AC, stage IV AC, stage IIIB SCC, stage IV SCC, and HC. Cluster 0 was characterized by high expressions in stage IIIB AC, stage IV AC, stage IIIB SCC, and a similar expression in stage IV SCC when compared with HC, with a peak expression in stage IV AC. This pattern was seen for 19 of the 73 genes. Cluster 1 was characterized by high expressions in stage IIIB AC, stage IV AC, stage IIIB SCC, and stage IV SCC when compared with HC, and a low expression in stage IIIB SCC alone when compared with stage IV AC, with a peak expression in stage IV AC. This pattern was seen for 16 of the 73 genes. Cluster 2 was characterized by high expressions in stage IIIB, stage IV AC, and stage IV SCC, and a similar expression in stage IIIB SCC when compared with HC. This pattern was the most common and seen for 20 of the 73 genes. Cluster 3 was also characterized by high expressions in stage IIIB, stage IV AC, and stage IV SCC, and a similar expression in stage IIIB SCC when compared with HC, but a higher intensity of relative gene expression in each gene compared with that in cluster 2. This pattern was seen for 18 of the 73 genes (Table S4). Among these 73 genes, ten overlapped with the 69 genes identified in the comparison between stage IIIB/IV cancer patients and the healthy controls: *Transforming growth factor beta 2* (*TGFB2*; modulation of proliferative activity, cellular differentiation, and embryological development), *Inhibin beta E* (*INHBE*; apoptotic function), *Platelet factor 4* (*PF4;* regulation of leukocytes trafficking ), *v-kit Hardy-Zuckerman 4 feline sarcoma viral oncogene homolog* (*KIT;* hematopoietic maintenance, growth, and differentiation ), *Protein tyrosine phosphatase receptor type N* (*PTPRN;* hormone and neuropeptide secretion), *Phospholipid scramblase 4* (*PLSCR4;* transbilayer movement of phospholipids across the plasma membrane), *Integrin beta 8* (*ITGB8;* TGF-beta activation), *S100A15*, *Complement component 1 q subcomponent C chain* (*C1QC;* triggering the complement classical pathway and augmenting phagocytosis), and *CRISP3*.

### Confirmation of the Microarray Results using TaqMan Real-time RT-PCR

**Figure 3:**
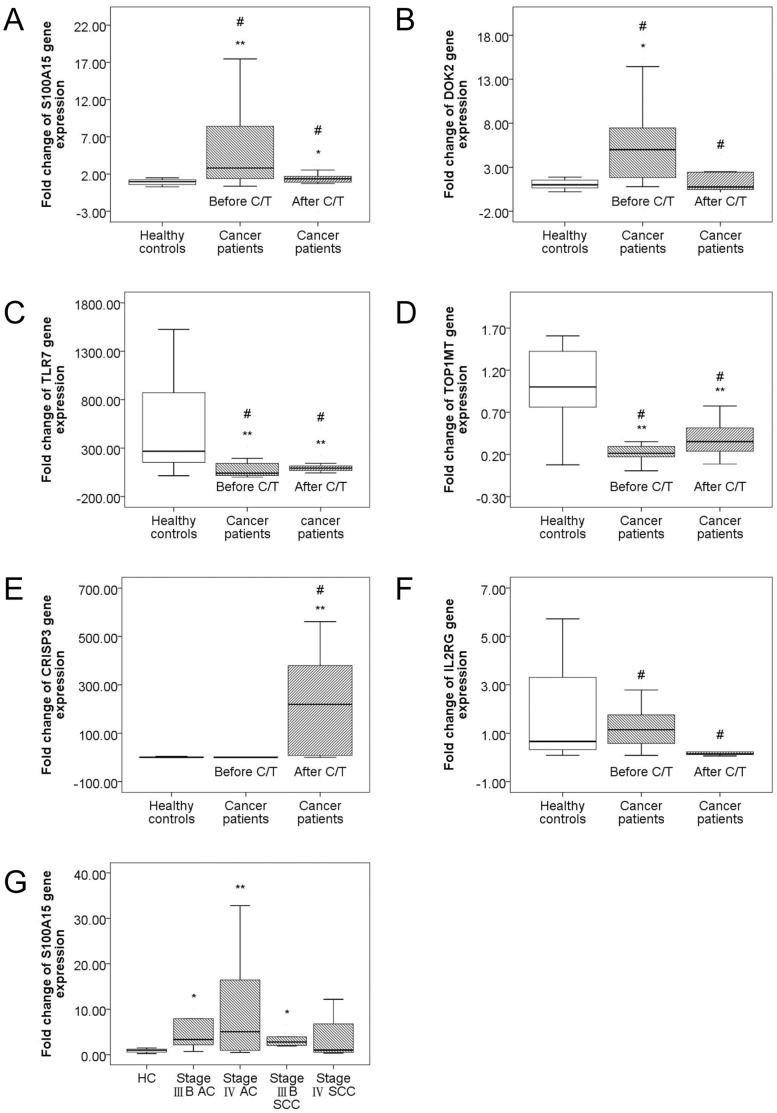
Confirmation of microarray results with TaqMan real-time RT-PCR. The box plots show the 25^th^, 50^th^, and 75^th^ percentiles, maximum, and minimum. For all cancer patients (striped box, N = 30) and healthy controls (HC, blank box, N = 20), expression levels of the six selected genes were measured using TaqMan real-time RT-PCR on the same RNA samples used for the microarrays. Median expression levels are shown for *S100A15* (A), *DOK2* (B), *TLR7* (C), *TOP1MT* (D), *CRISP3* (E), *IL2RG* (F). *S100A15* and *DOK2* were up-regulated in NSCLC patients compared to the HC, whereas *TLR7* and *TOP1MT* were down-regulated. *S100A15, DOK2*, and *IL2RG* were down-regulated after chemotherapy, whereas *TLR7*, *TOP1MT*, and *CRISP3* were up-regulated after chemotherapy. (G) Median expression level in parallel with the results of unsupervised hierarchical clustering is shown for *S100A15*, a cluster 1 gene with greater expression levels in stage IIIB AC, stage IIIB SCC, and stage IV AC compared to the HC, and a peak expression in stage IV AC. * p<0.05, compared with the healthy controls. ** p<0.01, compared with the healthy controls. # p<0.05, compared before and after chemotherapy.

To confirm the results obtained with the microarrays, the expression levels of six of the differentially expressed genes with respect to advanced stage lung cancer or chemotherapy treatment were assessed by an independent method of RNA quantification, TaqMan real-time RT-PCR, using the same RNA samples that were used for microarray analysis. Comparisons of the relative expression levels by real-time quantitative RT-PCR for the 4 genes that were up-regulated (*S100A15*, p = 0.001, Figure 3A; *DOK2*, p<0.001, Figure 3B) or down-regulated (*toll-like receptor 7* (*TLR7*; innate immunity), p<0.001, Figure 3C; *topoisomerase I mitochondrial* (*TOP1MT*; DNA Topoisomerase ATP-hydrolyzing activity), p = 0.005, Figure 3D) in the cancer patients compared with the HC, and the 6 genes that were up-regulated (*TLR7*, p = 0.015, Figure 3C; *TOP1MT,* p = 0.063, Figure 3D; CRISP3, p = 0.003, Figure 3E) or down-regulated (*S100A15*, p = 0.009, Figure 3A; *DOK2*, p = 0.007, Figure 3C; *IL2RG*, p = 0.001, Figure 3F) after chemotherapy compared with those before chemotherapy confirmed the validity of the microarray results. Through RT-PCR quantification, *S100A15* also showed a greater expression in stage IIIB AC, stage IIIB SCC, and stage IV AC compared to the HC, paralleling the results of supervised hierarchical clustering (cluster 1 gene) (Figure 3G).

### Protein Expressions of Lung Cancer Tissues by IHC Staining for the Differentially Expressed Genes Associated with Tumor Progression and/or Chemotherapy Signatures

**Figure 4:**
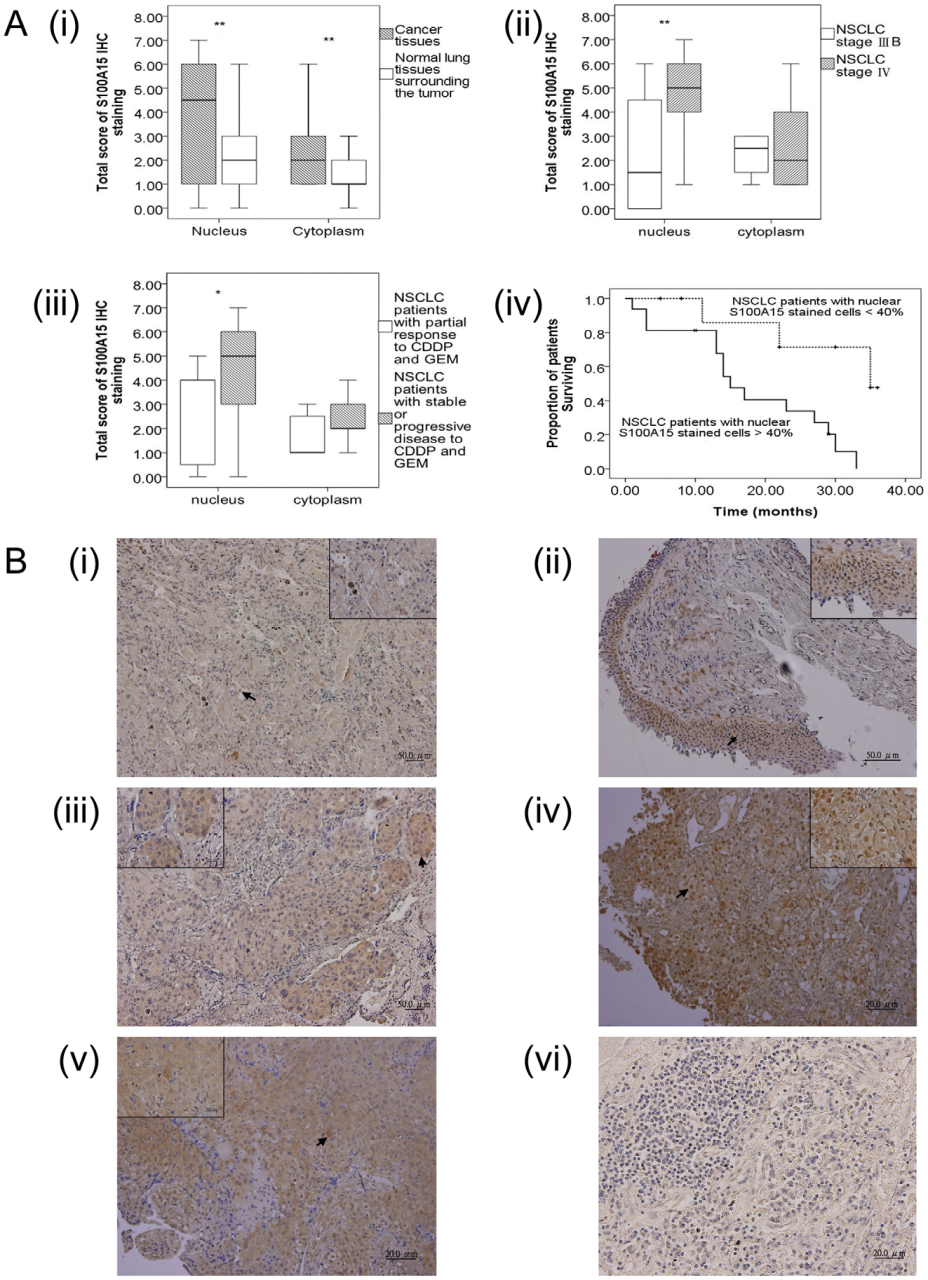
Immunohistochemistry (IHC) staining for S100A15. (A) Box-plot analysis by Mann-Whitney U test: The box plots show the distribution of nuclear and cytoplasmic total scores based on IHC of S100A15 protein in paraffin-embedded sections of cancer tissues and normal lung tissues surrounding the tumor. The box plots show the 25^th^, 50^th^, and 75^th^ percentiles, maximum, and minimum of the total immunostaining score (IHC scoring range 0–7). (i) Comparison of nuclear and cytoplasmic S100A15 expression between cancer tissues and normal lung tissues surrounding the tumor (N = 26) with a p value<0.01(**); (ii) Comparison of nuclear and cytoplasmic S100A15 expression between stage IIIB (N = 12) and stage IV (N = 14) non-small cell lung cancer with a p value<0.01(**); (iii) Comparison of nuclear and cytoplasmic S100A15 expression between patients with a partial response to combination chemotherapy (N = 7) with CDDP and GEM and those with stable or progressive disease (N = 19) with a p value <0.05(*). (iv) Kaplan-Meier survival curves for the 26 NSCLC patients with separate lines according to the percentage of nuclear S100A15 stained cells at a cut-off level of 40% showed significant differences in three-year survival rate (p = 0.008). (B) IHC analysis of S100A15 in paraffin-embedded sections of histologically proven adenocarcinoma (AC) and squamous cell carcinoma (SCC). (i) Normal lung tissues surrounding the tumor showing no nuclear and weak cytoplasmic S100A15 immunostaining; (ii) Stage IIIB AC showing scarce nuclear and weak cytoplasmic S100A15 immunostaining; (iii) Stage IIIB SCC showing weak nuclear and moderate cytoplasmic S100A15 immunostaining; (iv) stage IV AC depicting strong nuclear and moderate cytoplasmic S100A15 immunostaining; (v) stage IV SCC depicting strong nuclear and strong cytoplasmic S100A15 immunostaining; (vi) NSCLC used as a negative control showing no S100A15 immunostaining in the tumor cells, where the primary antibody was replaced by isotype specific non-immune mouse IgG. Arrows show nuclear and cytoplasmic localization (i-vi, original magnification × 100). Insets within the images show close-ups of the cells surrounding the arrows (I–V, original magnification × 400).

To determine the clinical significance of S100A15 protein in NSCLC tumorigenesis, we assessed the expression level by IHC staining using the paraffin embedded lung cancer tissues obtained at diagnosis from 26 of the same 30 cancer patients (Figure 4A, Figure 4B, and Table S5), whereas tissue sections of the other 4 patients were lost and unavailable. S100A15 could be detected weakly to strongly in tumor cells, very weakly in alveolar epithelial cells, and moderately in some macrophage, but not in lymphocytes. Figure 4A(i) shows the total immunostaining score distribution of nuclear/cytoplasmic S100A15 expression in the cancer tissues and normal lung tissues surrounding the tumors. Significant increases in both nuclear (p<0.001) and cytoplasmic (p = 0.002) S100A15 immunostaining of the cancer tissues were found compared to normal lung tissues surrounding the tumor. A significant increase in nuclear S100A15 expression was observed not only in stage IV NSCLC compared to stage IIIB NSCLC (p = 0.005), but also in patients with stable or progressive disease compared to those with a partial response (p = 0.032) (Figure 4A (ii and iii). Kaplan-Meier survival analysis showed significantly reduced three-year overall survival (p = 0.008 by the log-rank test; median survival 15 months) in the NSCLC patients harboring increased nuclear expressions of S100A15 (>40% nuclear stained cells), compared with median survival of 35 months in the patients showing weak nuclear *S100A15* staining (<40% nuclear stained cells) (Figure 4A (iv)). Forward stepwise multivariate Cox-regression analysis demonstrated that a high percentage of S100A15 nuclear stained cells (HR 1.028, 95% CI 1.007–1.05, p = 0.01) was the only independent factor associated with three-year overall mortality when adjusting for age, tumor stage, Eastern Cooperative Oncology Group performance status, histopathological subtype, smoking history, and co-morbidity (Table 3). However, neither nuclear (p = 0.87) nor cytoplasmic (p = 0.331) S100A15 staining showed an association with histopathological subtype. Quantitative RT-PCR result for *S100A15* was positively correlated with cytoplasmic S100A15 staining intensity score (Pearson’s correlation: r = 0.408, p = 0.038), but not with other parameters in IHC staining. Additionally, a search for the differentially expressed genes identified in this study to be associated with tumor progression and/or chemotherapy signatures was conducted in the Human Protein Atlas database (www.proteinatlas.com) [27]. This database contains annotated images and staining intensity from immunostained tissue microarrays for lung cancer and normal tissues. Among the 69 differentially expressed genes associated with tumor progression, 16 have been previously demonstrated to have increased protein expressions in lung cancer tissues, 19 decreased, and 14 similar compared with normal lung tissues in the Human Protein Atlas. Among the 59 differentially expressed genes associated with chemotherapy, 12 have been previously demonstrated to have increased protein expressions in lung cancer tissues, 9 decreased, and 15 similar compared with normal lung tissues in the Human Protein Atlas (Table 4).

**Table 3:** Use of Cox proportional hazard model to analyze predictors for 3-year overall mortality in the 26 non-small cell lung cancer patients.

	Bivariate		Multivariate	
	Harzard Ratio	95%ConfidenceInterval	Harzard Ratio	95%ConfidenceInterval
Age, years	1.013	0.973–1.054		
Male sex	0.854	0.279–2.612		
Tumor stage, IIIB versus IV	0.973	0.382–2.477		
Squamous cell carcinoma versus adenocarcinoma	0.48	0.189–1.221		
ECOG performance status	2.581	0.825–8.075		
Smoking history of more than 10 pack-years	6.402	1.502–27.296*		
Percentage of S100A15 nuclear stained cell, %	1.028	1.007–1.05*	1.028	1.007-1.05*
Nuclear S100A15 percentage positivity score	1.701	1.094–2.645*		
Nuclear S100A15 staining intensity score	1.547	1.013–2.363*		
Nuclear S100A15 total score	1.354	1.053–1.741*		
Percentage of S100A15 cytoplasmic stained cell, %	0.986	0.96–1.011		
Cytoplasmic S100A15 percentage positivity score	0.822	0.533–1.266		
Cytoplasmic S100A15 staining intensity score	0.31	0.099–0.975*		
Cytoplasmic S100A15 total score	0.779	0.541–1.12		
Diabetes mellitus	0.938	0.214–4.102		
Hypertension	1.151	0.443–2.99		
Chronic obstructive pulmonary disease	3.581	1.247–10–298*		
Chronic hepatitis	1.052	0.24–4.614		
Chronic kidney disease	0.042	0–118.7		
Congestive heart failure	1.807	0.232–14.053		

* p<0.05.

**Table 4:** Immunohistochemistry staining of lung cancer tissues compared with normal lung tissues published in the Human Protein Atlas website* for the differentially expressed genes identified in this study associated with tumor progression and/or chemotherapy signatures.

Increased#	Decreased#	Same#	Not expressed#
SLC22A16, TOP2A,EP300, NRG1, DUPD1,DAPP1, PTPRN2, PTPRT, PTPRZ1, ITGB8, S100A15, TIMP4, CCR2, BCL2L1, STAT1,CXCR4, PRKCZ, S100A8, MAPK14, BAD,SHC1, MAPRE1, TTK, STXBP1, TRAM1	SLC22A4, GNMT,ICMT, FIGF, BMP1,FGF3, CECR1, INHBE, PLSCR4, PLA2G4F, PLA2G10, SGMS2, CD1D, DDX58, TLR4,LXN, PF4, KIT, DOK2, PIK3CD, TLR5,BCL3, RASA1, ELK1, APBA1,STXBP2	HAT1, BDNF,PTPRN, PIK3C3, SERINC1, SPTLC1, C1QC, IL1RAP, DEFB118, CTNNA2,TIMP1, TIMP2, FES, FKBP5, IL4R, PLCG1, IFNB1, CD86, ATP6AP2, PTK2, CREBBP, PTPN6, KIF20A, CENPE, PRC1, MAP7, SMC1A, TUBB4, MAP3K11, UNC84B, RAB2A, COPB1, DOP2, TOP1MT, CARM1, MYST2, PTPRA, PTPRE	CEL, ACPP, INPP5D

* www.proteinatlas.com.

# Increased, decreased, the same, or not expressed in protein levels of lung cancer tissues, compared with that of normal lung tissues in the Human Protein Atlas.

## Discussion

Lung carcinogenesis is a complex process involving epithelial mesenchymal transition, which is an unregulated process in a host environment with deregulated inflammatory responses that impair both innate and adaptive immunity and permits cancer progression [2]. Although numerous gene expression prognostic signatures have been identified for NSCLC by cDNA microarrays in the last few years, most of these studies have focused on early stage cancers or responses to surgical therapy, and used tumor samples obtained before surgery for comparisons[15]–[24], [28]. In the current study, we identified *IL4* pathway-associated genes in immune cells that showed differential expressions between patients with advanced stage NSCLC and age-, sex-, and co-morbidity-matched healthy controls, some of which could be reverted or progressed after a median of four courses of combination chemotherapy with CDDP and GEM. Moreover, we identified and validated S100A15 to be a novel biomarker of tumor staging and a predictor of poor treatment response or long-term outcomes in advanced stage NSCLC.

Several different immunosuppressive cells, including myeloid derived suppressor cells (MDSC), tumor-associated macrophages (TAM), and T regulatory cells have shown increased populations in the cultured PBMC from cancer patients, implying an important mechanism of tumor immune evasion [29], [30], [31], [32]. In animal models, MDSC have been shown to impair tumor immunity by suppressing T cell activation and inducing TAM activation, thereby enhancing a tumor-promoting Th2 response [33], [34]. Chemotherapy treatment with doxorubicin plus cyclophosphamide in breast cancer patients has been shown to result in a decrease in the number of MDSC [32], and the Th2 response has been shown to partially reverse with GEM treatment in an animal model of mammary carcinoma [33]. These findings support our observation that the intersection of the PBMC gene expression signatures involved in tumor progression and chemotherapy results in *IL4* pathway-associated molecules, some of which were reversed after combination chemotherapy with CDDP and GEM in 17 of the 30 patients we examined.

IL4 binds to interleukin-4 receptor (IL4R) and IL2RG to trigger a Th2 predominant signaling pathway involving 49 discovered and other unknown genes. IL4 has been found to act as an autocrine survival factor in epithelial cells from colon, breast, and lung carcinomas [35]. In our study, both *IL4R* and *CXCR4* gene expressions were decreased with tumor progression, whereas both *DOK2* and *IL2RG* gene expressions were increased with tumor progression. In accordance with our result, Mazzoccoli et al reported that NSCLC was associated with a significant increase in serum IL2 levels, reflecting disease development [36]. Serum soluble IL-2R concentrations have been reported to be higher in patients with stage IIIB or IV NSCLC patients [37]. In contrast, CXCR4, which plays a pivotal role in the metastasis of NSCLC, has been reported to show an increased expression in tumor tissues [38]. Likewise, IL4R has been found to be expressed at higher levels in situ in lung tumor samples [39]. Furthermore, Kossenkov et al, using microarray to analyze gene expression in PBMC from early stage NSCLC patients, found that *CXCR4* showed higher expression before surgery compared to that after tumor removal, and was positively correlated with better survival [40]. In addition, *DOK2*, which is a downstream mediator of *IL2RG*, has been identified as a suppressor of lung cancer cell proliferation [41]. All of these three genes were up- or down-regulated in our PBMC samples in the opposite direction to the previous studies on lung tumor samples. Some differentially expressed genes associated with tumor progression identified in this study were up- or down-regulated in parallel with the IHC staining results published in the Human Protein Atlas website, whereas some were in the opposite direction (Table 3). With regards to this divergence, we speculate that the PBMC gene signatures from cancer patients may denote general immune responses mounted to combat the progression of in situ lung tumors, and may reflect the results of complex interactions between immunopotentiative and immunosuppressive cells. Moreover, all of the abnormal expressions of *CXCR4*, *DOK2*, and *IL2RG* in the PBMC were partly or totally reversed after the combination chemotherapy, implying a potential role of the *IL4* pathway in antitumor immunity.

Another three *IL4* pathway genes (*PRKCZ*, *PLCG1*, *PIK3CD*) that were down-regulated with tumor progression showed further decreased expressions after chemotherapy in the current study. PRKCZ has been shown to correlate with metastasis potential in pancreatic cancer cells [42], and PLCG1 and PIK3CD have been demonstrated to be associated with invasiveness of several cancer cells [43], [44]. In contrast, *STAT1*, which was up-regulated with tumor progression in our study, showed a further increase in expression after chemotherapy. Activation of the interferon/STAT1 pathway has been reported to correlate with resistance to radiotherapy, doxorubicin chemotherapy, and cetuximab target therapy in lung cancer [45], [46]. Taken together, this suggests that some immune surveillance functions involved in the *IL4* pathway are not regained after combination chemotherapy, and may contribute to resistance to treatment.

Another novel finding in our study is the up-regulation of *S100A15*, a calcium- and zinc-binding protein, in the PBMC from cancer patients, which also returned to normal after combination chemotherapy. Furthermore, we observed an increased nuclear S100A15 expression in lung cancer tissues not only in stage IV NSCLC compared to stage IIIB NSCLC, but also in the patients with stable or progressive disease in comparison to those with a partial response after first line combination chemotherapy with CDDP and GEM. Additionally, a high percentage of S100A15 nuclear stained cells was the only independent factor associated with three-year overall survival. This suggests that nuclear accumulation of S100A15 may be linked to metastasis potential, treatment response, and long-term outcomes. In accordance with the results published in the Human Protein Atlas website [27], we found up-regulation of S100A15 in NSCLC cancer tissues compared to normal lung tissues surrounding the tumor. Nonetheless, *S100A15* in either cancer tissues or PBMC could not distinguish AC from SCC in this small sample-size study. Additionally, quantitative RT-PCR result for *S100A15* was positively correlated with cytoplasmic S100A15 staining intensity score but not with other parameters in IHC staining assessment, implying that cytoplasmic protein expression rather than nuclear protein expression may affect *S100A15* expression in peripheral immune cells, such as monocyte. Disruption of the calcium signaling pathway, such as by the S100 family, has been implicated as a central mechanism in tumorigenesis, specifically tumor invasion and metastasis [47]. Both S100A15 (also called S100A7A) and S100A7 proteins have been demonstrated to be distinctly expressed in normal breast tissue and breast cancer [48]. Since S100A15 was found to be chemotactic for both granulocytes and monocytes, and to act synergistically with highly homologous S100A7 to enhance inflammation [49], [50], both proteins could influence lung tumor progression. Additionally, *E. coli* can modulate the human S100A15 expression of keratinocytes by recognition through TLR4, suggesting that S100A15 may play a role in innate immunity [51]. Selective expression of S100A7 in lung SCC and large cell carcinomas has been demonstrated, but not in AC or small cell carcinomas [52]. Nuclear accumulation of S100A7 has been reported to be associated with a poor prognosis in head and neck cancer [26]. To the best of our knowledge, the current study is the first to demonstrate that nuclear accumulation of S100A15 may be linked to an increased risk of distant metastasis, and that S100A15 may serve as a candidate biomarker for predicting treatment response and survival. However, large scale longitudinal studies are warranted to evaluate the potential of S100A15 as a determinant of advanced tumor stage and/or a predictor of long-term outcomes in NSCLC.

Apart from *S100A15*, we also found that several innate immunity-associated genes were differentially expressed between cancer patients and the HC. In our study, the *TLR7* gene was down-regulated in cancer patients according to the RT-PCR results, and partially returned to normal levels after combination chemotherapy. In a previous microarray gene expression study by Showe et al, *TLR1, 5, 7,* and *8* were all down-regulated with concomitant suppression of the *NFκB* pathway in PBMC samples from early stage NSCLC patients, suggesting that innate response pathways are suppressed in the general immune response to NSCLC [23]. In another microarray study on PBMC from NSCLC patients, significant elevation in gene expression levels for *TLR5, 6, 7, 8,* and *10* was found after surgery as compared to that before tumor removal [53]. Stimulation with TLR7 agonists on human lung cancer cells has been shown to lead to increased tumor cell survival and chemoresistance [54]. On the other hand, systemic administration of TLR7 agonists has also been found to induce significant antitumor activity, which could be potentiated by cyclophosphamide [55]. In a cell culture model, TLR7 agonists were found to enhance tumor cell lysis by human gamma delta T cells [56]. Taken together, these findings suggest that enhancing the TLR7 expression in immune cells may potentiate the anti-tumor effect of combination chemotherapy in advanced stage NSCLC patients. In contrast with the results of early stage lung cancer reported by Showe et al [23], we found that both *TLR1* and *TLR4* were up-regulated with tumor progression, and that *TLR5* was up-regulated only after chemotherapy. This suggests that innate immune responses may be somewhat different between early and advanced stage NSCLC patients.

Another five genes that were up-regulated with tumor progression showed further increased expressions after chemotherapy (*CRISP3, KCNMA1, BDNF, SLC22A4, SYN2*). CRISP3 and KCNMA1 have been demonstrated to be correlated with metastasis potential in prostate cancer and breast cancer, respectively [57], [58]. BDNF has been shown to promote anti-apoptotic proteins of lung AC and several other cancer cells [59], [60]. These three genes may be candidate biomarkers to predict tumor progression and response to chemotherapy. SLC22A4, which is an organic cation transporter and has been previously identified to confer cellular uptake and sensitivity to anti-tumor drugs [61], may play a role in the pharmacokinetics of CDDP and GEM. The *Syn2* gene has been implicated in synaptogenesis, neurotransmitter release, and the localization of nitric oxide synthase [62]. The relationship between *Syn2* and cancer has not yet been reported and needs to be clarified in future studies.

The present study is biased and has several limitations. First, we used PBMC rather than immune cells in cancer tissues to represent the tumor-induced alteration of the immune system. Genetic signatures from PBMC may be dynamically influenced by blood-based immune effector cells, primary tumor, metastatic tumor, and rare tumor cells occasionally detected in blood from cancer patients. However, gene expression profiles derived from PBMC have been suggested as a promising tool for the early detection or prediction of prognosis in cancer patients in several previous studies, implying that a similar regulation of genes may be present in immune cells of cancer tissues and peripheral blood from cancer patients [23], [63]. Second, the sample size of this study is relatively small, particularly for survival analysis. However, we estimated the power to be 87% for the comparison of real time RT-PCR *S100A15* gene expression levels in PBMC between the patients with NSCLC and the HC, and 95% for the comparison of nuclear S100A15 total immunostaining scores in lung cancer tissues between stage IIIB and IV patients using a two-sided Mann-Whitney test, and assuming that the actual distribution was logistic with a simple adjustment to the sample size in our study and an α error of 0.05 with PASS 2005 (NCSS, Kaysville, Utah, USA) software. On the other hand, a Cox regression of the log hazard ratio on percentage of S100A15 nuclear stained cells with a standard deviation of 28.1% based on a sample of 26 observations achieves 69% power at a 0.05 significance level to detect a regression coefficient equal to 0.028, with adjustment for an event rate of 0.327. Third, although the *IL4* pathway was significantly enriched in both the tumor progression and chemotherapy signatures, the *IL4* gene was not differentially expressed in either of the two comparisons, and the expression levels of two receptors for *IL4* (*IL4R* and *IL2RG*) were divergent. The underlying mechanisms by which the *IL4* pathway was involved in the immune surveillance of NSCLC tumorigenesis needs to be investigated further. Finally, only patients with advanced stage AC or SCC were enrolled in this study, and the results may not be applicable to early stage NSCLC or the large/small cell carcinoma subtype.

In summary, we found gene expression signatures in PBMC that were differentially expressed between patients with advanced stage NSCLC and healthy controls, as well as before and after chemotherapy. The intersection of tumor progression and chemotherapy signatures in immune cells suggest that the *IL4* pathway may play a potential role in the immune surveillance of NSCLC tumorigenesis, and immune potentiation of combination chemotherapy for NSCLC. Moreover, we identified and validated *S100A15, DOK2, TLR7, IL2RG, CRISP3,* and *TOP1MT* as candidate biomarkers, which may provide an important mechanistic understanding of immune response regulation in patients with advanced stage NSCLC receiving first line combination chemotherapy. Finally, nuclear S100A15 accumulation in lung cancer tissues may be associated with a greater distant metastatic potential, poor response to chemotherapy, and poor long-term survival in patients with NSCLC.

## Supporting Information

Table S1 Primer sequences and probe product number for assay quantitative real-time polymerase chain reactions used in the present study.
(DOC)

Table S2 Selected microarray gene expression significantly altered in peripheral blood mononuclear cells (PBMC) of advanced stage non-small cell lung cancer patients compared with healthy subjects.
(DOC)

Table S3 Selected microarray gene expression significantly altered in PBMC of advanced-stage non-small cell lung cancer patients after chemotherapy with cisplatin and gemcitabine compared with that before chemotherapy.
(DOC)

Table S4 Selected microarray gene expression significantly up-regulated in PBMC of advanced stage non-small cell lung cancer patients with different stages and histopathologies in association with 1.5 fold change identified by unsupervised hierarchical clustering analysis.
(DOC)

Table S5 Raw data of immunohistochemical staining results for *S100A15*.
(DOC)
