# Supplementary material for: Peripheral Immune Cell Gene Expression Changes in Advanced Non-Small Cell Lung Cancer Patients Treated with First Line Combination Chemotherapy
Source: PLoS One. 2013 Feb 25;8(2):e57053. doi: 10.1371/journal.pone.0057053 (PMC3581559; doi:10.1371/journal.pone.0057053)
Supplement: Table S1 — Primer sequences and probe product number for assay quantitative real-time polymerase chain reactions used in the present study. (DOC) [file pone.0057053.s001.doc]

**Table S1.** Primer sequences and probe product number for assay quantitative real-time polymerase chain reactions used in the present study.

| Gene name |  | Primer sequences | Probe product number |
| --- | --- | --- | --- |
| *S100A15* | Forward | 5'-tctcactcatccttctactcgtga | #48 |
|  | Reverse | 5'-tcagcttgagtgttgctcatc |  |
| *TLR7* | Forward | 5'-ccagtgtctaaagaacctggaaac | #5 |
|  | Reverse | 5'-tcagggacagtggtcagttg |  |
| *TOP1MT* | Forward | 5'-gccctgtatttcatcgataagc | #15 |
|  | Reverse | 5'-gcctcaccgtcctccttc |  |
| *CRISP3* | Forward | 5'-ctggaaaccactgcaatgac | #48 |
|  | Reverse | 5'-gcagtaaaagcgggatcctt |  |
| *DOK2* | Forward | 5'-gaaacaaggcttcttgtatcttcag | #84 |
|  | Reverse | 5'-gtccgaccctccatacagtg |  |
| *IL2RG* | Forward | 5'-tgctaaaactgcagaatctggt | #33 |
|  | Reverse | 5'-caagaatctgttgttccagttca |  |

# <https://www.roche-applied-science.com/>
