# Supplementary material for: Peripheral Immune Cell Gene Expression Changes in Advanced Non-Small Cell Lung Cancer Patients Treated with First Line Combination Chemotherapy
Source: PLoS One. 2013 Feb 25;8(2):e57053. doi: 10.1371/journal.pone.0057053 (PMC3581559; doi:10.1371/journal.pone.0057053)
Supplement: Table S2 — Selected microarray gene expression significantly altered in peripheral blood mononuclear cells (PBMC) of advanced stage non-small cell lung cancer patients compared with healthy subjects. (DOC) [file pone.0057053.s002.doc]

**Table S2. Selected microarray gene expression significantly altered in peripheral blood mononuclear cells (PBMC) of advanced stage non-small cell lung cancer patients compared with healthy subjects.**

| **Gene Name** | | **Fold change** | | | | **Genbank** | | | **Description** | | |
| --- | --- | --- | --- | --- | --- | --- | --- | --- | --- | --- | --- |
|  | **Stage**  **ⅢB** | | | **Stage Ⅳ** | |  | | | | |  |
| **Secretion** | | | | | | | | | | | |
| CEL | | | 3.830 | | 5.379 | NM_001807.3 | | | Carboxyl ester lipase (bile salt-stimulated lipase) (CEL) | | |
| LTBP4 | | | 1.926 | | 1.184 | NM_001042545.1 | | | Latent transforming growth factor beta binding protein 4 (LTBP4), transcript variant 3 | | |
| KCNMA1 | | | 1.512 | | 3.606 | NM_002247.2 | | | Potassium large conductance calcium-activated channel, subfamily M, alpha member 1 (KCNMA1), transcript variant 2 | | |
| SLC22A4 | | | 1.538 | | 1.712 | NM_003059.2 | | | Solute carrier family 22 (organic cation transporter), member 4 (SLC22A4) | | |
| SYN2 | | | 4.078 | | 6.393 | NM_133625.3 | | | Synapsin II (SYN2), transcript variant IIa | | |
| SLC22A16 | | | 1.415 | | 2.339 | NM_033125.2 | | | Solute carrier family 22 (organic cation transporter), member 16 (SLC22A16) | | |
| **DNA Topoisomerase Activity** | | | | | | | | | | | |
| TOP2A | | | 2.369 | | 2.574 | NM_001067.2 | | | Topoisomerase (DNA) II alpha 170kDa | | |
| **Methyltransferase Activity** | | | | | | | | | | | |
| GNMT | | | 1.301 | | 1.586 | | NM_018960.4 | | | | Glycine N-methyltransferase |
| ICMT | | | 2.007 | | 3.462 | | NM_170705.1 | | | | Isoprenylcysteine carboxyl methyltransferase, transcript variant 2 |
| **Histone Acetyltransferase Activity** | | | | | | | | | | | |
| CDY1B | | | 2.409 | | 4.537 | | NM_001003894.1 | | | | Chromodomain protein, Y-linked, 1B |
| HAT1 | | | 1.350 | | 1.590 | | NM_003642.2 | | | | Histone acetyltransferase 1, transcript variant 1 |
| EP300 | | | 1.295 | | 1.519 | | NM_001429.2 | | | | E1A binding protein p300 |
| **Growth Factor Activity** | | | | | | | | | | | |
| FIGF | | | 2.596 | | 7.103 | | NM_004469.2 | | | | c-fos induced growth factor (vascular endothelial growth factor D) |
| TGFB2 | | | 2.205 | | 15.49 | | NM_003238.1 | | | | Transforming growth factor, beta 2 |
| BMP1 | | | 1.534 | | 2.001 | | NM_006131.1 | | | | Bone morphogenetic protein 1, transcript variant BMP1-5 |
| FGF3 | | | 1.539 | | 2.483 | | NM_005247.2 | | | | Fibroblast growth factor 3 |
| IL9 | | | 3.301 | | 5.562 | | NM_000590.1 | | | | Interleukin 9 |
| CECR1 | | | 1.467 | | 2.019 | | NM_177405.1 | | | | cat eye syndrome chromosome region, candidate 1, transcript variant 2 |
| INHBE | | | 3.022 | | 6.280 | | NM_031479.3 | | | | Inhibin, beta E |
| NRG1 | | | 2.215 | | 4.833 | | NM_013960.2 | | | | Neuregulin 1, transcript variant ndf43 |
| BDNF | | | 1.255 | | 1.565 | | NM_001709.3 | | | | Brain-derived neurotrophic factor (BDNF), transcript variant 4 |
| **Protein Tyrosine Phosphatase Activity** | | | | | | | | | | | |
| ACPP | | | 2.117 | | 3.054 | | NM_001099.2 | | | | Acid phosphatase, prostate |
| DUPD1 | | | 2.204 | | 4.033 | | NM_001003892.1 | | | | Dual specificity phosphatase and pro isomerase domain containing 1 |
| DAPP1 | | | 1.464 | | 1.838 | | NM_014395.1 | | | | Dual adaptor of phosphotyrosine and 3-phosphoinositides |
| PTPRN2 | | | 1.394 | | 1.847 | | NM_130842.1 | | | | Protein tyrosine phosphatase, receptor type, N polypeptide 2, transcript variant 2 |
| PTPRT | | | 1.987 | | 3.960 | | NM_007050.5 | | | | Protein tyrosine phosphatase, receptor type, T, transcript variant 2 |
| PTPRZ1 | | | 1.925 | | 3.254 | | NM_002851.2 | | | | Protein tyrosine phosphatase, receptor-type, Z polypeptide 1 |
| PTPRN | | | 2.003 | | 4.187 | | NM_002846.2 | | | | Protein tyrosine phosphatase, receptor type, N |
| EYA1 | | | 4.937 | | 10.26 | | NM_172060.1 | | | | Eyes absent homolog 1 (Drosophila), transcript variant 1 |
| **Membrane Lipid Metabolic Process** | | | | | | | | | | | |
| PLSCR4 | | | 2.778 | | 4.518 | | NM_020353.1 | | | | Phospholipid scramblase 4 |
| PLA2G4F | | | 2.039 | | 3.395 | | NM_213600.2 | | | | Phospholipase A2, group IVF |
| ITGB8 | | | 2.717 | | 7.498 | | NM_002214.2 | | | | Integrin, beta 8 |
| PIK3C3 | | | 1.264 | | 1.566 | | NM_002647.2 | | | | Phosphoinositide-3-kinase, class 3 |
| PLA2G10 | | | 2.044 | | 2.840 | | NM_003561.1 | | | | Phospholipase A2, group X |
| SGMS2 | | | 2.632 | | 4.558 | | NM_152621.4 | | | | Sphingomyelin synthase 2 |
| SERINC1 | | | 2.107 | | 2.487 | | NM_020755.2 | | | | Serine incorporator 1 |
| SPTLC1 | | | 1.338 | | 1.677 | | NM_178324.1 | | | | Serine palmitoyltransferase, long chain base subunit 1, transcript variant 2 |
| **Innate Immune Response** | | | | | | | | | | | |
| S100A15 | | | 1.794 | | 3.658 | | NM_176823.3 | | | | S100 calcium binding protein A15(A7A) |
| C1QB | | | 1.984 | | 3.506 | | NM_000491.3 | | | | Complement component 1, q subcomponent, B chain |
| C1QC | | | 3.649 | | 7.950 | | NM_172369.2 | | | | Complement component 1, q subcomponent, C chain |
| CD1D | | | 1.554 | | 1.999 | | NM_001766.3 | | | | T-cell surface glycoprotein CD1d Precursor (CD1d antigen) |
| CRISP3 | | | 2.067 | | 3.273 | | NM_006061.1 | | | | Cysteine-rich secretory protein 3 |
| IL1RAP | | | 1.947 | | 2.670 | | NM_002182.2 | | | | Interleukin 1 receptor accessory protein, transcript variant 1 |
| DDX58 | | | 1.312 | | 1.622 | | NM_014314.3 | | | | DEAD (Asp-Glu-Ala-Asp) box polypeptide 58 |
| NOD2 | | | 1.374 | | 1.611 | | NM_022162.1 | | | | Nucleotide-binding oligomerization domain containing 2 |
| TLR1 | | | 1.478 | | 1.854 | | NM_003263.3 | | | | Toll-like receptor 1 |
| TLR4 | | | 1.566 | | 2.208 | | NM_138554.2 | | | | Toll-like receptor 4 |
| DEFB118 | | | 1.798 | | 2.897 | | NM_054112.1 | | | | Defensin, beta 118 |
| **Cell adhesion molecule binding** | | | | | | | | | | | |
| CTNNAL1 | | | 2.408 | | 3.546 | | NM_003798.2 | Catenin (cadherin-associated protein), alpha-like 1 (CTNNAL1) | | | |
| CTNNA2 | | | 5.613 | | 9.001 | | NM_004389.2 | Catenin (cadherin-associated protein), alpha 2 (CTNNA2) | | | |
| **Metalloendopeptidase Inhibitor Activity** | | | | | | | | | | | |
| LXN | | | 1.316 | | 1.512 | | NM_020169.2 | Latexin | | | |
| TIMP1 | | | 1.505 | | 1.806 | | NM_003254.2 | TIMP metallopeptidase inhibitor 1 | | | |
| TIMP2 | | | 1.291 | | 1.738 | | NM_003255.4 | TIMP metallopeptidase inhibitor 2 | | | |
| TIMP4 | | | 1.685 | | 2.634 | | NM_003256.2 | TIMP metallopeptidase inhibitor 4 | | | |
| **Cytokine and Chemokine Mediated Signaling Pathway** | | | | | | | | | | | |
| PF4 | | | 3.648 | | 7.655 | | NM_002619.1 | | | Platelet factor 4 (chemokine (C-X-C motif) ligand 4) | |
| KIT | | | 7.089 | | 16.30 | | NM_001093772.1 | | | v-kit Hardy-Zuckerman 4 feline sarcoma viral oncogene homolog, transcript variant 2 | |
| CCR1 | | | 1.704 | | 2.409 | | NM_001295.2 | | | Chemokine (C-C motif) receptor 1 | |
| CCR2 | | | 1.589 | | 2.051 | | NM_000647.3 | | | Chemokine (C-C motif) receptor 2 | |
| **IL-4 Pathway** | | |  | |  | |  | | |  | |
| ***Up-***  ***regulated*** | | |  | |  | |  | | |  | |
| IL2RG | | | 2.631 | | 1.950 | | NM_000206.1 | | | Interleukin 2 receptor, gamma | |
| BCL2L1 | | | 2.207 | | 1.938 | | NM_138578.1 | | | BCL2-like 1 (BCL2L1), nuclear gene encoding mitochondrial protein, transcript variant 1 | |
| FES | | | 2.081 | | 2.402 | | NM_002005.2 | | | Feline sarcoma oncogene | |
| DOK2 | | | 1.585 | | 1.500 | | NM_003974.2 | | | Docking protein 2, 56kDa | |
| FKBP5 | | | 1.475 | | 1.407 | | NM_004117.2 | | | FK506 binding protein 5 | |
| STAT1 | | | 1.156 | | 1.800 | | NM_007315.2 | | | Signal transducer and activator of transcription 1, 91kDa, transcript variant alpha | |
| ***Down-***  ***regulated*** | | |  | |  | |  | | |  | |
| CXCR4 | | | 0.165 | | 0.186 | | NM_003467.2 | | | Chemokine (C-X-C motif) receptor 4 (CXCR4), transcript variant 2 | |
| IL4R | | | 0.426 | | 0.241 | | NM_001008699.1 | | | Interleukin 4 receptor (IL4R), transcript variant 2 | |
| PRKCZ | | | 0.664 | | 0.307 | | NM_002744.4 | | | Protein kinase C, zeta (PRKCZ), transcript variant 1 | |
| IL4R | | | 0.748 | | 0.587 | | NM_000418.2 | | | Interleukin 4 receptor (IL4R), transcript variant 1 | |
| PLCG1 | | | 0.748 | | 0.492 | | NM_182811.1 | | | Phospholipase C, gamma 1 (PLCG1), transcript variant 2 | |
| PIK3CD | | | 0.764 | | 0.690 | | NM_005026.2 | | | Phosphoinositide-3-kinase, catalytic, delta polypeptide | |
