# Supplementary material for: Peripheral Immune Cell Gene Expression Changes in Advanced Non-Small Cell Lung Cancer Patients Treated with First Line Combination Chemotherapy
Source: PLoS One. 2013 Feb 25;8(2):e57053. doi: 10.1371/journal.pone.0057053 (PMC3581559; doi:10.1371/journal.pone.0057053)
Supplement: Table S3 — Selected microarray gene expression significantly altered in PBMC of advanced-stage non-small cell lung cancer patients after chemotherapy with cisplatin and gemcitabine compared with that before chemotherapy. (DOC) [file pone.0057053.s003.doc]

**Table S3. Selected microarray gene expression significantly altered in PBMC of advanced-stage non-small cell lung cancer patients after chemotherapy with cisplatin and gemcitabine compared with that before chemotherapy.**

| **Gene Name** | **Fold change** | **Genbank** | **Description** |
| --- | --- | --- | --- |
| **Innate Immune Response** | | | |
| ***Up-regulated*** | | | |
| CRISP3 | 3.781 | NM_006061.1 | Cysteine-rich secretory protein 3 |
| IFNB1 | 4.166 | NM_002176.2 | Interferon, beta 1 |
| TLR5 | 2.047 | NM_003268.4 | Toll-like receptor 5 |
| TLR7 | 1.547 | NM_016562.3 | Toll-like receptor 7 |
| CD86 | 1.123 | NM_006889.3 | CD86 molecule, transcript variant 2, T lymphocyte activation B7-2 antigen |
| ***Down-regulated*** | | | |
| S100A8 | 0.604 | NM_002964.3 | S100 calcium binding protein A8 |
| S100A15 | 0.376 | NM_172863.3 | S100 calcium binding protein A15(A7A) |
| **Cytokine Production** | | | |
| ***Up-regulated*** | | | |
| ATP6AP2 | 1.645 | NM_005765.2 | Renin receptor Precursor (ATPase H(+)-transporting lysosomal accessory protein 2) |
| NLRC4 | 1.530 | NM_021209.3 | NLR family, CARD domain containing 4 |
| ***Down-regulated*** | | | |
| BCL3 | 0.445 | NM_005178.3 | B-cell CLL/lymphoma 3 |
| **IL-4 Pathway** | | | |
| ***Up-regulated*** | | | |
| CXCR4 | 1.451766 | NM_003467.2 | Homo sapiens chemokine (C-X-C motif) receptor 4 (CXCR4), transcript variant 2, mRNA. |
| MAPK14 | 2.456007 | NM_139012.1 | Homo sapiens mitogen-activated protein kinase 14 (MAPK14), transcript variant 2, mRNA. |
| STAT1 | 1.602298 | NM_007315.2 | Homo sapiens signal transducer and activator of transcription 1, 91kDa (STAT1), transcript variant alpha, mRNA. |
| PTK2 | 1.433014 | NM_005607.3 | Homo sapiens PTK2 protein tyrosine kinase 2 (PTK2), transcript variant 2, mRNA. |
| BAD | 2.96357 | NM_032989.1 | Homo sapiens BCL2-antagonist of cell death (BAD), transcript variant 2, mRNA. |
| RASA1 | 2.347742 | NM_022650.1 | Homo sapiens RAS p21 protein activator (GTPase activating protein) 1 (RASA1), transcript variant 2, mRNA. |
| PRKCZ | 2.467911 | NM_001033581.1 | Homo sapiens protein kinase C, zeta (PRKCZ), transcript variant 2, mRNA. |
| ***Down-regulated*** | | | |
| PRKCZ | 0.421248 | NM_002744.4 | Homo sapiens protein kinase C, zeta (PRKCZ), transcript variant 1, mRNA. |
| CREBBP | 0.756314 | NM_001079846.1 | Homo sapiens CREB binding protein (Rubinstein-Taybi syndrome) (CREBBP), transcript variant 2, mRNA. |
| DOK2 | 0.718981 | NM_003974.2 | Homo sapiens docking protein 2, 56kDa (DOK2), mRNA. |
| PTPN6 | 0.65738 | NM_080548.3 | Homo sapiens protein tyrosine phosphatase, non-receptor type 6 (PTPN6), transcript variant 2, mRNA. |
| PTPN6 | 0.335961 | NM_080549.2 | Homo sapiens protein tyrosine phosphatase, non-receptor type 6 (PTPN6), transcript variant 3, mRNA. |
| SHC1 | 0.389466 | NM_183001.3 | Homo sapiens SHC (Src homology 2 domain containing) transforming protein 1 (SHC1), transcript variant 1, mRNA. |
| SHC1 | 0.726735 | NM_003029.3 | Homo sapiens SHC (Src homology 2 domain containing) transforming protein 1 (SHC1), transcript variant 2, mRNA. |
| INPP5D | 0.794359 | NM_005541.3 | Homo sapiens inositol polyphosphate-5-phosphatase, 145kDa (INPP5D), transcript variant 2, mRNA. |
| IL2RG | 0.436801 | NM_000206.1 | Homo sapiens interleukin 2 receptor, gamma (severe combined immunodeficiency) (IL2RG), mRNA. |
| PLCG1 | 0.500105 | NM_182811.1 | Homo sapiens phospholipase C, gamma 1 (PLCG1), transcript variant 2, mRNA. |
| PIK3CD | 0.625551 | NM_005026.2 | Homo sapiens phosphoinositide-3-kinase, catalytic, delta polypeptide (PIK3CD), mRNA. |
| ELK1 | 0.40967 | NM_005229.2 | Homo sapiens ELK1, member of ETS oncogene family (ELK1), mRNA. |
| **Microtubule-based Process** | | | |
| ***Up-regulated*** | | | |
| KIF20A | 3.441 | NM_005733.1 | Kinesin family member 20A |
| CENPE | 2.791 | NM_001813.2 | Centromere protein E, 312kDa |
| PRC1 | 1.871 | NM_199413.1 | Protein regulator of cytokinesis 1, transcript variant 2 |
| APBA1 | 2.864 | NM_001163.2 | Amyloid beta (A4) precursor protein-binding, family A, member 1 |
| MAPRE1 | 1.374 | NM_012325.1 | Microtubule-associated protein, RP/EB family, member 1 |
| MAP7 | 2.252 | NM_003980.3 | Microtubule-associated protein 7 |
| TTK | 2.181 | NM_003318.3 | Phosphotyrosine picked threonine-protein kinase |
| ***Down-regulated (spindle organization and biogenesis)*** | | | |
| SMC1A | 0.484 | NM_006306.2 | Structural maintenance of chromosomes 1A |
| TUBB4 | 0.203 | NM_006087.2 | Tubulin, beta 4 |
| TUBB4Q | 0.722 | NM_020040.3 | Tubulin, beta polypeptide 4, member Q |
| MAP3K11 | 0.661 | NM_002419.3 | Mitogen-activated protein kinase kinase kinase 11 |
| UNC84B | 0.569 | NM_015374.1 | Unc-84 homolog B (C. elegans) |
| **Organic Cation Transmembrane Transporter Activity** | | | |
| ***Up-regulated*** | | | |
| SLC22A4 | 1.726 | NM_003059.2 | solute carrier family 22 (organic cation transporter), member 4 |
| RHAG | 4.244 | NM_000324.1 | Rh-associated glycoprotein |
| **Secretory Pathway** | | | |
| ***Up-regulated (synaptic transmission)*** | | | |
| SYN2 | 4.632 | NM_133625.3 | Synapsin II, transcript variant IIa |
| BDNF | 4.023 | NM_001709.3 | Brain-derived neurotrophic factor, transcript variant 4 |
| CADPS2 | 3.067 | NM_017954.9 | Ca2+-dependent activator protein for secretion 2, transcript variant 1 |
| KCNMA1 | 2.028 | NM_002247.2 | Potassium large conductance calcium-activated channel, subfamily M, alpha member 1 |
| STXBP1 | 1.727 | NM_003165.1 | Syntaxin binding protein 1, transcript variant 1 |
| TRAM1 | 1.543 | NM_014294.4 | Translocation associated membrane protein 1 |
| RAB2A | 1.679 | NM_002865.1 | Member RAS oncogene family, Ras-related protein Rab-2A |
| COPB1 | 1.186 | NM_016451.3 | Coatomer protein complex, subunit beta 1 |
| COPB2 | 1.589 | NM_004766.1 | Coatomer protein complex, subunit beta 2 |
| ***Down-regulated*** | | | |
| STXBP2 | 0.715 | NM_006949.1 | Syntaxin binding protein 2 (Unc-18 homolog 2) |
| DOP2 | 0.535 | NM_005128.2 | Dopey family member 2 |
| SRPR | 0.710 | NM_003139.2 | Signal recognition particle receptor ('docking protein') |
| **DNA Topoisomerase (ATP-hydrolyzing) Activity** | | | |
| ***Up-regulated*** | | | |
| TOP1MT | 1.730 | NM_052963.1 | Topoisomerase (DNA) I, mitochondrial, nuclear gene encoding mitochondrial protein |
| **Histone Methyltransferase Activity** | | | |
| ***Up-regulated*** | | | |
| MLL3 | 1.816 | NM_170606.2 | Myeloid/lymphoid or mixed-lineage leukemia 3, transcript variant 2 |
| ***Down-regulated*** | | | |
| CARM1 | 0.526 | NM_199141.1 | Coactivator-associated arginine methyltransferase 1 |
| EHMT2 | 0.610 | NM_025256.4 | Euchromatic histone-lysine N-methyltransferase 2, transcript variant NG36/G9a-SPI |
| **Histone Acetyltransferase Activity** | | | |
| ***Down-regulated*** | | | |
| MYST2 | 0.582 | NM_007067.3 | MYST histone acetyltransferase 2 |
| **Protein Tyrosine Phosphatase Activity** | | | |
| ***Down-regulated*** | | | |
| PTPRA | 0.316 | NM_080840.2 | Protein tyrosine phosphatase, receptor type, A, transcript variant 2 |
| PTPRE | 0.662 | NM_130435.2 | Protein tyrosine phosphatase, receptor type, E, transcript variant 2 |
