# Supplementary material for: Peripheral Immune Cell Gene Expression Changes in Advanced Non-Small Cell Lung Cancer Patients Treated with First Line Combination Chemotherapy
Source: PLoS One. 2013 Feb 25;8(2):e57053. doi: 10.1371/journal.pone.0057053 (PMC3581559; doi:10.1371/journal.pone.0057053)
Supplement: Table S4 — Selected microarray gene expression significantly up-regulated in PBMC of advanced stage non-small cell lung cancer patients with different stages and histopathologies in association with 1.5 fold change identified by unsupervised hierarchical clustering analysis. (DOC) [file pone.0057053.s004.doc]

**Table S4. Selected microarray gene expression significantly up-regulated in PBMC of advanced stage non-small cell lung cancer patients with different stages and histopathologies in association with 1.5 fold change identified by unsupervised hierarchical clustering analysis**

| **Gene Name** | **Cluster** | **Genbank** | **Description** |
| --- | --- | --- | --- |
| ***Significantly Associated GO Terms in Cluster 0*** | | | |
| **Lipid Transporter Activity** | | | |
| PON1 | 0 | NM_000446.3 | Paraoxonase 1 |
| PLSCR4 | 0 | NM_020353.1 | Phospholipid scramblase 4 |
| **Response to Wounding** | | | |
| TGFB2 | 0 | NM_003238.1 | Transforming growth factor, beta 2 |
| FUT10 | 0 | NM_032664.3 | Fucosyltransferase 10 (alpha (1,3) fucosyltransferase) |
| PLSCR4 | 0 | NM_020353.1 | Phospholipid scramblase 4 |
| NOX4 | 0 | NM_016931.2 | NADPH oxidase 4 |
| **Innate Immune Response** | | | |
| C1QC | 0 | NM_172369.2 | Complement component 1, q subcomponent, C chain |
| COLEC12 | 0 | NM_130386.1 | Collectin sub-family member 12 |
| *(Genes in other cluster)* | | | |
| S100A15 | 1 | NM_176823.3 | S100 calcium binding protein A15(A7A) |
| **Isomerase Activity** | | | |
| PPIC | 0 | NM_000943.4 | Peptidylprolyl isomerase C (cyclophilin C) |
| PPIL4 | 0 | NM_139126.2 | Peptidylprolyl isomerase (cyclophilin)-like 4 |
| **Steroid Metabolic Process** | | | |
| NPC1L1 | 0 | NM_013389.1 | NPC1 (Niemann-Pick disease, type C1, gene)-like 1 |
| NR1I2 | 0 | NM_003889.3 | Nuclear receptor subfamily 1, group I, member 2, transcript variant 1 |
| UGT2B17 | 0 | NM_001077.2 | UDP glucuronosyltransferase 2 family, polypeptide B17 |
| *(Genes in other cluster)* | | | |
| STS | 1 | NM_000351.4 | Steroid sulfatase (microsomal), isozyme S, steroid metabolism |
| PBX1 | 1 | NM_002585.1 | Pre-B-cell leukemia homeobox 1 |
| HMGCS2 | 2 | NM_005518.2 | 3-hydroxy-3-methylglutaryl-Coenzyme A synthase 2 (mitochondrial) |
| **Receptor Activity** | | | |
| KIT | 0 | NM_001093772.1 | v-kit Hardy-Zuckerman 4 feline sarcoma viral oncogene homolog, hemopoiesis |
| P2RX3 | 0 | NM_002559.2 | Purinergic receptor P2X, ligand-gated ion channel, 3 |
| PLA2R1 | 0 | NM_001007267.1 | Phospholipase A2 receptor 1, 180kDa, transcript variant 2 |
| OPRL1 | 0 | NM_182647.1 | Opiate receptor-like 1 (OPRL1), transcript variant 1 |
| ***Significantly Associated GO Terms in Cluster 1*** | | | |
| **Growth Factor Activity** | | | |
| FGF7 | 1 | NM_002009.2 | Fibroblast growth factor 7 (keratinocyte growth factor) |
| FGF20 | 1 | NM_019851.1 | Fibroblast growth factor 20 |
| **Anion Transmembrane Transporter Activity** | | | |
| SLCO1A2 | 1 | NM_134431.2 | Solute carrier organic anion transporter family, member 1A2, transcript variant 1 |
| SLC17A4 | 1 | NM_005495.1 | Solute carrier family 17 (sodium phosphate), member 4 |
| **Lipid catabolic process** | | | |
| PLA2GA4 | 1 | NM_024420.2 | Phospholipase A2, group IVA (cytosolic, calcium-dependent) |
| STS | 1 | NM_000351.4 | Steroid sulfatase (microsomal), isozyme S |
| **Chromosome Centromeric Region** | | | |
| TIGD1 | 1 | NM_145702.1 | Tigger transposable element derived 1 |
| SGOL2 | 1 | NM_152524.3 | Shugoshin-like 2 (S. pombe) |
| **Neurofilament Cytoskeleton** | | | |
| GAN | 1 | NM_022041.2 | Giant axonal neuropathy (gigaxonin) |
| **Extracellular Matrix** | | | |
| FBN2 | 1 | NM_001999.3 | Fibrillin 2 (congenital contractural arachnodactyly) |
| CRISP3 | 1 | NM_006061.1 | Cysteine-rich secretory protein 3 |
| FLRT2 | 1 | NM_013231.4 | Fibronectin leucine rich transmembrane protein 2 |
| TFIP11 | 1 | NM_001008697.1 | Tuftelin interacting protein 11, transcript variant 1 |
| *(Genes in other cluster)* | | | |
| ADAMTS20 | 3 | NM_025003.3 | ADAM metallopeptidase with thrombospondin type 1 motif, 20 |
| FRAS1 | 3 | NM_025074.4 | Fraser syndrome 1 |
| ***Significantly Associated GO Terms in Cluster 2***  **Signal Transducer Activity** | | | |
| PTGFR | 2 | NM_001039585.1 | Prostaglandin F receptor (FP), transcript variant 2 |
| MYLK | 2 | NM_053032.2 | Myosin, light chain kinase, transcript variant 8 |
| TNK1 | 2 | NM_003985.1 | Tyrosine kinase, non-receptor, 1 |
| ITGB8 | 2 | NM_002214.2 | Integrin, beta 8 |
| PTPRN | 2 | NM_002846.2 | Protein tyrosine phosphatase, receptor type, N |
| TREML4 | 2 | NM_198153.1 | Triggering receptor expressed on myeloid cells-like 4 |
| GNG12 | 2 | NM_018841.4 | Guanine nucleotide binding protein (G protein), gamma 12 |
| FOLR1 | 2 | NM_016731.2 | Folate receptor 1 (adult), transcript variant 8 |
| **Myosin Binding** | | | |
| CALD1 | 2 | NM_033140.2 | caldesmon 1, transcript variant 5 |
| **Protein Tetramerization** | | | |
| TP73L | 2 | NM_003722.3 | Tumor protein p73-like |
| **DNA Replication** | | | |
| NFIB | 2 | NM_005596.2 | nuclear factor I/B |
| MLH3 | 2 | NM_014381.2 | MutL homolog 3, transcript variant 2 |
| **Negative Regulation of Cell Differentiation** | | | |
| PF4 | 2 | NM_002619.1 | Platelet factor 4 (chemokine (C-X-C motif) ligand 4) |
| CNTN4 | 2 | NM_175607.1 | Contactin 4, transcript variant 1 |
| **Symporter Activity** | | | |
| SLC17A2 | 2 | NM_005835.1 | Slute carrier family 17 (sodium phosphate), member 2 |
| SLC6A15 | 2 | NM_018057.4 | Slute carrier family 6, member 15 transcript variant 2 |
| **Glucuronosyltransferase Activity** | | | |
| UGT2B10 | 2 | NM_001075.3 | UDP glucuronosyltransferase 2 family, polypeptide B10 |
| **Hormone Activity** | | | |
| TSHB | 2 | NM_002218.3 | Thyroid stimulating hormone, beta |
| INHBE | 2 | NM_031479.3 | Inhibin, beta E |
| *(Genes in other cluster)* | | | |
| INSL6 | 3 | NM_007179.2 | Insulin-like 6 |
| ***Significantly Associated GO Terms in Cluster 3*** | | | |
| **Homophilic Cell Adhesion** | | | |
| PCDHB15 | 3 | NM_018935.2 | Protocadherin beta 15 |
| PCDHB16 | 3 | NM_020957.1 | Protocadherin beta 16 |
| PCDH7 | 3 | NM_002589.2 | Protocadherin 7, transcript variant a |
| *(Genes in other cluster)* | | | |
| CDH20 | 0 | NM_031891.2 | Cadherin 20, type 2 |
| PCDH17 | 0 | NM_001040429.2 | Protocadherin 17 |
| **Ion Homeostasis** | | | |
| RHAG | 3 | NM_000324.1 | Rh-associated glycoprotein |
| SLC9A4 | 3 | NM_001011552.3 | Slute carrier family 9 (sodium/hydrogen exchanger), member 4 |
| **Steroid Hormone Receptor Binding** | | | |
| FHL2 | 3 | NM_201557.2 | Fur and a half LIM domains 2, transcript variant 4 |
| **Metal Ion Binding** | | | |
| TRIM4 | 3 | NM_033091.1 | Tipartite motif-containing 4, transcript variant beta |
| GATA1 | 3 | NM_002049.2 | GATA binding protein 1 (globin transcription factor 1) |
| PLOD2 | 3 | NM_000935.2 | Pocollagen-lysine, 2-oxoglutarate 5-dioxygenase 2, transcript variant 2 |
| CAPS2 | 3 | NM_032606.2 | Clcyphosine 2 |
| ZSCAN20 | 3 | NM_145238.3 | Znc finger and SCAN domain containing 20 |
| ZNF648 | 3 | NM_001009992.1 | Znc finger protein 648 |
| FRAS1 | 3 | NM_025074.4 | Fraser syndrome 1 |
| ADAMTS20 | 3 | NM_025003.3 | ADAM metallopeptidase with thrombospondin type 1 motif, 20 |
| **Electron transport Chain** | | | |
| NDUFC2 | 3 | NM_004549.3 | NADH dehydrogenase (ubiquinone) 1, subcomplex unknown, 2, 14.5kDa |
