# Supplementary material for: Peripheral Immune Cell Gene Expression Changes in Advanced Non-Small Cell Lung Cancer Patients Treated with First Line Combination Chemotherapy
Source: PLoS One. 2013 Feb 25;8(2):e57053. doi: 10.1371/journal.pone.0057053 (PMC3581559; doi:10.1371/journal.pone.0057053)
Supplement: Table S5 — Raw data of immunohistochemical staining results for S100A15. (DOC) [file pone.0057053.s005.doc]

**Table S5. Raw data of immunohistochemical staining results for *S100A15*.**

| Sample number | Tumor stage | Histo  pathology | Response to C/T | Time to progression, month | Percentage of nuclear stained cell, % | Nuclear staining intensity score | Percentage of cytoplasmic stained cell, % | Cytoplasmic staining intensity score |
| --- | --- | --- | --- | --- | --- | --- | --- | --- |
| 5 | Ⅲb | AC | SD | 18 | 42.12 | 3 | 8.40 | 2 |
| 6 | Ⅳ | AC | SD | 7 | 44.42 | 3 | 7.02 | 1 |
| 7 | Ⅲb | AC | SD | 4 | 53.72 | 3 | 13.51 | 2 |
| 8 | Ⅳ | AC | SD | 15 | 26.43 | 2 | 7.35 | 1 |
| 9 | Ⅲb | AC | PR | 6 | 7.38 | 0 | 2.35 | 1 |
| 11 | Ⅳ | SCC | PR | 5 | 59.38 | 1 | 4.52 | 1 |
| 12 | Ⅳ | AC | SD | 15 | 82.37 | 3 | 11.07 | 1 |
| 15 | Ⅳ | AC | PD | 6 | 52.45 | 3 | 5.36 | 1 |
| 17 | Ⅲb | SCC | SD | 4 | 0.0 | 0 | 6.50 | 1 |
| 18 | Ⅲb | SCC | PD | 9 | 16.34 | 0 | 19.52 | 2 |
| 21 | Ⅳ | SCC | PR | 8 | 67.54 | 1 | 10.70 | 1 |
| 25 | Ⅳ | AC | SD | 6 | 79.92 | 3 | 12.69 | 1 |
| 28 | Ⅳ | AC | PD | 4 | 60.96 | 2 | 31.64 | 1 |
| 29 | Ⅳ | AC | PD | 13 | 19.40 | 0 | 85.40 | 2 |
| 31 | Ⅲb | AC | PR | 13 | 12.61 | 0 | 79.50 | 2 |
| 32 | Ⅲb | AC | PR | 5 | 43.23 | 2 | 13.90 | 2 |
| 10 | Ⅳ | SCC | PD | 1 | 70.36 | 2 | 85.70 | 1 |
| 13 | Ⅳ | AC | SD | 4 | 86.36 | 2 | 37.91 | 2 |
| 14 | Ⅳ | AC | PD | 2 | 38.04 | 3 | 13.50 | 3 |
| 16 | Ⅲb | AC | SD | 26 | 6.42 | 0 | 25.67 | 2 |
| 19 | Ⅲb | SCC | SD | 9 | 43.44 | 3 | 18.47 | 1 |
| 20 | Ⅲb | SCC | PD | 2 | 51.67 | 1 | 15.14 | 1 |
| 24 | Ⅲb | AC | PR | 2 | 1.18 | 0 | 4.40 | 1 |
| 26 | Ⅲb | AC | SD | 5 | 7.09 | 1 | 19.55 | 2 |
| 27 | Ⅳ | SCC | SD | 3 | 90.00 | 3 | 23.04 | 1 |
| 34 | Ⅳ | SCC | PR | 12 | 66.48 | 2 | 7.31 | 1 |

C/T = chemotherapy; AC = adenocarcinoma; SCC = squamous cell carcinoma; PR = partial response; SD = stable disease; PD = progressive disease
